# A modular, multi-sensor crawler robot for adaptive pipeline inspection: design and experimental validation

**DOI:** 10.1038/s41598-025-32719-y

**Published:** 2026-01-08

**Authors:** Ahmed A. Abd Eltwab, Ahmed Sameh

**Affiliations:** 1 Department of Mechatronics Engineering, Faculty of Engineering, Mansoura University, Mansoura, Egypt; 2 Department of Production Engineering and Mechanical Design, Faculty of Engineering, Mansoura University, Mansoura, Egypt

**Keywords:** In-pipe inspection, Crawler robot, Autonomous navigation, Real-time defect detection, Raspberry Pi, Ultrasonic sensing, Gas leak detection, Structural health monitoring, Energy science and technology, Engineering

## Abstract

The increasing age of petroleum pipeline infrastructure poses significant risks to safety, operational efficiency, and the environment. Defects such as cracks, corrosion, joint displacement, and deformation remain major causes of leakage and catastrophic failure. Traditional inspection approaches rely heavily on manually operated robotic crawlers, with defect detection dependent on human review of video footage, resulting in time-consuming and error-prone assessments. This paper presents the design, fabrication, and experimental validation of an autonomous modular crawler robot integrating a Raspberry Pi 4, Arduino Mega, high-resolution camera, ultrasonic distance sensors, and gas detection capabilities for real-time, multi-modal defect detection in petroleum pipelines. The proposed system achieves autonomous navigation, real-time video streaming, and multi-sensor data fusion, enabling robust inspection in varying pipe diameters and material conditions. Laboratory and simulated field experiments demonstrated a maximum speed of 0.25 m/s, obstacle detection accuracy of 91.2%, climb capability of up to 45°, and battery endurance of approximately 80 min. Compared to existing inspection systems, the proposed crawler robot offers improved adaptability, sensing integration, and autonomy. The results position the system as a viable solution for preventive pipeline maintenance, with potential extensions into AI-driven defect classification and SLAM-based navigation.

## Introduction

Petroleum pipelines are vital components of modern infrastructure, enabling the continuous transport of crude oil, natural gas, and refined products over vast distances. According to the U.S. Pipeline and Hazardous Materials Safety Administration (PHMSA), corrosion and material failure accounted for over 25% of significant pipeline incidents between 2010 and 2023, causing extensive economic and environmental damage^1,2^. Globally, many pipeline networks have exceeded their intended design life, increasing the likelihood of structural deterioration^3–6^.

Common defects include longitudinal and transverse cracks, pitting corrosion, weld seam failures, and mechanical deformation^7–12^. These defects, if undetected, can lead to hazardous leaks, explosions, and service interruptions. Timely inspection and preventive maintenance are essential to mitigate such risks. However, conventional inspection relies heavily on tethered or remotely operated crawlers, where human operators visually interpret recorded footage. This approach is slow, labor-intensive, and susceptible to oversight, particularly for subtle or partially obscured damage^13,14^.

Recent advances in robotics and sensing technologies have yielded more capable inspection solutions, such as Pipeline Inspection Gauges (PIGs), wheeled crawlers, and snake-like articulated robots^15–20^. Nevertheless, these systems still face **three key challenges**:

1. Limited adaptability to varying pipe diameters and geometries.
2. Dependence on human control and interpretation.
3. Lack of integrated, real-time multi-sensor data fusion for automated decision-making.

This research addresses these challenges by presenting a fully functional prototype of a modular, autonomous crawler robot capable of real-time defect detection in petroleum pipelines. The system incorporates high-resolution visual inspection, ultrasonic obstacle detection, and gas leakage sensing in a compact, corrosion-resistant chassis, with wireless data transmission for remote monitoring.

### Novelty distinction from prior work

Although recent in-pipe inspection platforms such as Jeon et al. (2024) and Lucet et al. (2024) have advanced the state of modular mechanical designs and sensing packages, our system introduces a set of technical innovations that are distinct from these works. First, unlike Jeon et al. whose robot operates primarily in a semi-autonomous mode and is optimized for large-diameter water pipelines, the proposed crawler is designed for **fully onboard**,** real-time autonomy** within **150–200 mm pipelines**, relying on an integrated sensor suite (ultrasonic, vision, and gas) and a **lightweight edge-level fusion method** that enables obstacle detection and navigation decisions without continuous operator supervision. Second, compared with Lucet et al., who emphasize a teleoperated and modular architecture validated in field settings, our system achieves **adaptive mechanical compliance** across the targeted diameter range without hardware reconfiguration, while maintaining **low-cost sensing and processing** suited for routine inspection tasks. Third, our design explicitly prioritizes **cost–performance optimization**, achieving competitive detection accuracy and robustness in both PVC and steel environments using affordable components. In summary, the novelty of this work lies in combining **real-time onboard autonomy**, **diameter adaptability**, and **multi-sensor edge fusion** into a practical, low-cost inspection platform not previously demonstrated in the recent literature.

## Literature review

The past decade has seen rapid development in in-pipe and sewer inspection robots, ranging from high-end non-destructive testing (NDT) platforms to lightweight visual and ultrasonic crawlers. However, most prior works emphasize descriptive improvements in sensing or mobility without achieving a balanced combination of autonomy, adaptability, and multi-sensor fusion suitable for small-diameter pipelines (150–200 mm)^21–23^. This section provides a critical synthesis of prior research by comparing sensing modalities, locomotion strategies, autonomy levels, and their practical limitations, and highlights how the proposed system addresses these gaps.

### Locomotion and mechanical adaptability

Four main locomotion classes dominate the literature: wheeled crawlers, tracked platforms, magnetic clamping robots, and soft/adaptive mechanisms. Wheeled crawlers are energy-efficient but vulnerable to traction loss in oily or corroded steel pipes^24,25^. Tracked robots improve stability but significantly increase power consumption^26^. Magnetic adhesion systems provide excellent stability in steel pipes but cannot operate in PVC networks and usually support only one diameter range. Soft and articulated designs offer better adaptability to diameter variations but sacrifice payload capacity and mechanical simplicity^27,28^. A key limitation common to many prior systems is their narrow adaptability range—several platforms are optimized for a fixed diameter or require mechanical reconfiguration before deployment. This limits usability in municipal sewage and water networks where diameters vary along the same pipeline section.

### Sensing modalities

Existing inspection robots employ a wide range of sensing technologies depending on their target applications. Vision-based systems are widely used due to their rich semantic information and compatibility with AI-based defect classification^29^. However, they remain highly sensitive to lighting variation, water droplets, lens contamination, and specular reflections inside metallic pipes. Ultrasonic sensors, on the other hand, offer robust short-range obstacle detection even in complete darkness but provide limited contextual information and are prone to noisy readings on rough or oily surfaces. High-end electromagnetic methods such as Magnetic Flux Leakage (MFL) and eddy-current sensing deliver high-precision metal-loss detection^30–32^ but often require large pipe diameters, heavy payloads, and specialist interpretation pipelines, making them unsuitable for low-cost and small-diameter deployments. Many recent platforms rely on single-modality sensing, which leads to poor generalization across varying environments^33^.

### Autonomy and localization

The majority of earlier inspection robots operate under teleoperation, relying heavily on human visual assessment^34^. While teleoperation provides safety assurance, it restricts scalability and introduces latency. Semi-autonomous systems integrate simple obstacle avoidance but still depend on frequent operator intervention^35,36^. More recent research explores SLAM-enabled inspection, using LiDAR, stereo vision, or inertial measurement units. However, SLAM approaches struggle inside narrow pipes due to low texture, repetitive geometries, and limited sensor field-of-view. Additionally, SLAM-capable platforms are often expensive and power-hungry, making them impractical for routine inspections. Critically, few studies demonstrate consistent **onboard autonomy** without external computation or tether-based assistance in small-diameter pipes.

### AI-based defect recognition (2023–2025)

Recent trends show rapid progress in using convolutional neural networks, transformers, and lightweight edge architectures for automated crack detection, corrosion classification, and deposit segmentation^37–39^. Studies from 2023 to 2025 highlight promising accuracy improvements but also note major bottlenecks such as small datasets, domain shifts between pipe materials, and difficulty handling partial occlusions or low-light conditions. Most existing AI pipelines operate offline (post-processing), while only a few explore real-time or near-real-time inference on embedded platforms. Furthermore, AI models in prior work often lack synchronized multimodal inputs, relying solely on RGB imagery, which limits robustness in harsh sewer environments.

### Identified gaps in prior literature

A critical synthesis of the above reveals three persistent shortcomings in existing research:

1. Limited autonomy — most systems rely on teleoperation or partial automation without fully onboard decision-making.
2. Narrow adaptability — few platforms function reliably across the 150–200 mm diameter range without mechanical reconfiguration.
3. Lack of multimodal, time-synchronized fusion — most work uses either vision or ultrasonic sensing independently rather than fusing complementary modalities at the edge.
4. Experimental narrowness — limited evaluations under varying surface conditions (oily, corroded steel), material types (PVC, steel), and obstacle geometries.

### How the proposed work addresses these gaps

The system presented in this study directly targets these limitations through:

- Multi-sensor fusion at the edge combining ultrasonic ranging, RGB imaging, and gas sensing in a time-synchronized pipeline, enabling reliable obstacle detection even when one modality is degraded.
- A mechanically adaptive crawler capable of operating across 150–200 mm diameter pipes without hardware reconfiguration, addressing a key limitation of prior fixed-diameter systems.
- Onboard, real-time autonomy, where obstacle detection, navigation decisions, and data processing are performed locally without continuous operator involvement.
- Experimental validation across both PVC and steel pipes, including conditions such as corrosion and surface contamination, which are typically under-reported in earlier studies.
- A cost-performance oriented design that provides practical deployability for routine pipeline inspection, complementing—but not competing directly with—high-cost NDT and SLAM-heavy systems.

This expanded and analytical review positions the proposed robot as a practical contribution that bridges the gap between high-end, specialized inspection systems and low-cost platforms that historically lacked autonomy, adaptability, and robust sensing integration.

### Comparative analysis

Table 1 presents a comparison of representative in-pipe inspection robots from literature, highlighting their locomotion type, adaptability, sensing capabilities, autonomy level, and respective pros and cons.

**Table 1:** Comparative analysis of representative in-pipe inspection robots.

Year	System/reference	locomotion type	Pipe size adaptability	Sensing modalities	Autonomy level	Pros	Cons	Limitations
2016	Deepak et al.^24^	Inchworm	Low–Medium	Camera	Semi-autonomous	Good maneuverability; precise positioning	Slow speed; complex actuation	Limited diameter range; unsuitable for long pipelines
2018	PiROB^27^	Tracked crawler	Medium	Camera, NDT	Semi-autonomous	High traction in hazardous environments	High weight; limited flexibility	High energy consumption; limited adaptability
2020	Xu et al.^25^	Wheeled crawler	High	Camera	Teleoperated	Lightweight; high speed	Poor adaptability to sharp bends	No autonomous features
2022	Lucet et al.^28^	Modular crawler	High	Camera, ultrasonic	Teleoperated	Modular design; field-tested	Requires skilled operators; no autonomy	No real-time onboard fusion
2023	Tan et al.^37^	Snake-like	High	Camera, gas sensor	Semi-autonomous	Excellent bend negotiation; multi-sensor	Complex control; higher energy use	Mechanically complex
2024	Jeon et al.^38^	Wheeled crawler	High	Camera, MFL	Semi-autonomous	Strong NDT integration; adaptable wheels	Costly sensors; moderate speed	Medium autonomy; moderate speed
**This work**	—	Modular wheeled crawler	High	Camera, ultrasonic, gas sensors	**Full Autonomous navigation**	Low cost, Multi-modal sensing; adaptable; validated prototype	Wi-Fi signal loss in metallic pipes	**Wireless signal loss in steel pipelines**

## Petroleum pipeline systems and inspection requirements

Efficient design and deployment of robotic inspection systems require a clear understanding of petroleum pipeline structures, operational conditions, and the nature of defects that commonly occur. The **geometry**,** material composition**,** and functional requirements** of pipelines dictate both the mechanical and sensing capabilities that an inspection robot must possess^40,41^.

### Pipeline geometry and configurations

Petroleum pipeline networks vary in diameter, cross-sectional profile, and overall layout. In **trunk lines**, diameters may range between 1000 mm and 2000 mm, enabling high-volume transport of crude oil or refined products, while **distribution lines** can be as narrow as 500 mm^42^. The **circular cross-section** remains the industry standard, providing optimal pressure distribution and ease of manufacturing^43^. However, variations such as **spiral-welded**, **rectangular**, **coiled tubing**, and **clad pipes** are also used depending on application-specific needs.

- Circular (round) pipes: Offer uniform stress distribution and minimal hydraulic losses, making them ideal for high-pressure applications^44^.
- Spiral-welded pipes: Common in large-diameter, low- to medium-pressure applications; their weld seam can pose localized weakness points^45^.
- Rectangular or square cross-sections: Rare in petroleum transport; mostly used in structural frameworks or low-pressure containment^46^.
- Coiled tubing: Used extensively in well intervention and workover operations; requires inspection tools with high bending capability^47^.
- Clad or lined pipes: Designed for corrosive environments, with an inner corrosion-resistant layer such as stainless steel or polymer^48^.

The operational layout often includes **junctions**,** elbows**,** reducers**,** and branch lines**, which present additional navigational complexity for inspection robots. The ability to negotiate such configurations is a defining factor in robotic system design^49^.

### Operational conditions and their impact on inspection

Petroleum pipelines may be located **underground**,** underwater**,** or aboveground**, each presenting unique inspection challenges. Underground metallic pipelines often exhibit severe signal attenuation for wireless communications, necessitating hybrid or tethered data transmission solutions^50^. Underwater pipelines require enhanced sealing, buoyancy compensation, and resistance to biofouling^51^. Aboveground lines face greater risks from mechanical impacts, UV degradation, and temperature cycling^52^.

Internal conditions can vary significantly:

- Product-filled pipelines: Limit visual inspection; require NDT methods such as magnetic flux leakage or ultrasonic wall thickness measurement^53^.
- Empty/dry pipelines: Allow optical imaging but may still contain corrosive residues or debris.
- High-pressure pipelines: Impose strict safety regulations during inspection, often requiring robots to operate in partial-flow conditions^54^.

### Common defect types

Defects in petroleum pipelines can be classified into **mechanical**, **corrosion-related**, and **manufacturing-related** categories^55,56^.

- Corrosion defects: Include uniform corrosion, pitting corrosion, and stress corrosion cracking (SCC). Pitting corrosion is particularly hazardous because it can progress through the pipe wall with little external indication^57^.
- Cracks: Longitudinal cracks typically result from hoop stress, while transverse cracks often occur due to bending or ground movement. Weld defects are also common initiation points for crack propagation^58^.
- Mechanical damage: Caused by third-party interference (excavation strikes), vibration-induced fatigue, or improper installation. Dents and gouges can significantly reduce pipeline burst strength^59^.
- Hydrogen-induced cracking (HIC) and sulfide stress cracking (SSC): These are prevalent in sour service pipelines and require specialized detection methods^60^.

The detection of these defects is crucial not only for immediate safety but also for implementing predictive maintenance strategies. Industry standards such as **API 1163** and **ASME B31.8 S** provide guidelines for inspection frequency, defect classification, and maintenance planning^61,62^.

### Implications for robotic inspection system design

The diversity in pipe geometries, materials, and defect types imposes several design requirements on inspection robots:

1. Locomotion flexibility to handle varying diameters and geometries without loss of traction or stability.
2. Multi-modal sensing combining optical imaging, ultrasonic measurement, and gas detection for comprehensive defect characterization^63^.
3. Environmental robustness to withstand high humidity, corrosive atmospheres, and temperature extremes.
4. Data transmission reliability in both metallic and non-metallic pipelines, potentially using hybrid wired/wireless architectures^64^.
5. Autonomous navigation capable of obstacle avoidance and path optimization in complex pipeline networks^65^.

These operational realities underscore the importance of the **multi-sensor modular crawler design** proposed in this work, which was developed to specifically address these varied inspection demands.

## System design

The proposed crawler robot was designed to satisfy the diverse operational requirements of petroleum pipeline inspection, as discussed in Sect. 3. The design followed a **systems engineering approach**, incorporating mechanical, electronic, and control subsystems in a modular architecture to facilitate adaptability, ease of maintenance, and scalability for different pipeline environments^66^. A major design goal was to ensure **full operational autonomy** for routine inspection tasks while maintaining the ability to revert to manual teleoperation in complex or emergency situations.

### Design philosophy and approach

The system architecture was guided by three primary principles:

1. Modularity – enabling reconfiguration for various pipeline diameters without a complete redesign of the chassis.
2. Multi-sensor integration – combining visual, ultrasonic, and gas sensing for comprehensive defect detection.
3. Environmental resilience – ensuring the robot can withstand conditions typical in petroleum pipelines, such as high humidity, corrosion, and debris.

The design process followed the **V-model methodology** for mechatronic system development, starting with requirements definition, progressing through subsystem design and integration, and concluding with validation testing^67^.

### Mechanical design

**Fig. 1:**
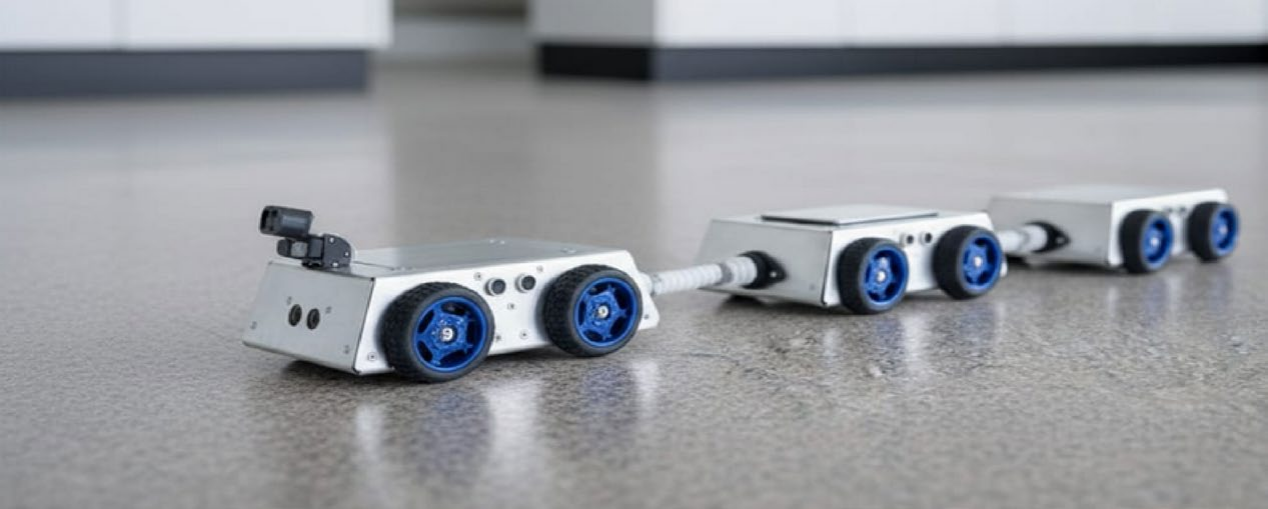
Prototype photo.

The realized prototype of the modular inspection robot is shown in Fig. 1. This physical prototype demonstrates three connected modules with articulated joints and forms the basis for subsequent design descriptions.

#### Chassis material selection

The chassis is constructed from **2 mm-thick 6061-T6 aluminum sheet**. This material was selected based on its **high strength-to-weight ratio** (yield strength ≈ 275 MPa, density 2.7 g/cm³), **excellent corrosion resistance**, and ease of machining^68^. Stainless steel was considered but rejected due to its higher density, which would negatively impact mobility in inclined pipelines. Composite materials such as carbon fiber were deemed cost-prohibitive for large-scale deployment.

The aluminum components were precision-cut using CNC laser processing to ensure dimensional accuracy and repeatability. A powder-coated finish was applied to further enhance corrosion resistance in chemically aggressive environments^69^.

#### Structural configuration

The base frame supports **four DC geared motors** mounted symmetrically to distribute load evenly and maintain stability. The wheel assemblies are located to maximize contact surface area while minimizing rolling resistance. The main body houses the sensor suite — including the Raspberry Pi camera, ultrasonic modules, and gas sensor — in a forward-facing configuration to optimize detection range and accuracy.

The **robot height** was carefully determined to allow clearance inside pipe diameters as small as 150 mm while accommodating all onboard components. A low center of gravity design was adopted to prevent tipping during sharp turns or while climbing inclined sections.

#### Wheel design and traction

Each wheel is **85 mm in diameter** and made from a composite material with a rubberized tread pattern. This configuration provides an optimal balance between **traction**,** durability**,** and shock absorption**. The wheel hubs are connected to the motor shafts via precision-machined aluminum couplers to minimize misalignment and power losses. To further improve grip in contaminated environments, interchangeable tread covers with higher friction coefficients can be installed for specific missions^70^.

#### Articulation mechanism

To navigate T-junctions, elbows, and other complex geometries, the robot uses **articulated joints** between modular sections. These joints employ high-torque servo actuators capable of ± 45° rotation, allowing the robot to pivot and align itself with branch lines without losing traction^71^. The joint housings are sealed to prevent ingress of dust, oil, or water.

### Electronics and sensor suite

The electronic subsystem integrates **control**,** sensing**,** and communication modules** into a compact and robust layout. The design emphasizes low power consumption to maximize operational runtime, as well as **redundancy** to prevent mission failure in the event of single sensor or component malfunction.

The main processing tasks — such as image acquisition, real-time video streaming, and multi-sensor data fusion — are handled by a **Raspberry Pi 4 Model B** with 4 GB RAM. This single-board computer offers sufficient computational power for onboard image processing and communication management^72^. Low-level motor control, sensor interfacing, and PWM speed regulation are managed by an **Arduino Mega 2560**, which communicates with the Raspberry Pi via a USB serial link. The sensors used are as follows.

- Raspberry Pi camera module v2: Captures 8 MP images at 30 fps, with a horizontal field of view (FOV) of 62.2°, suitable for internal pipe inspection^73^.
- Ultrasonic sensors (HC-SR04): Positioned at the front and flanks to provide obstacle detection up to 30 cm with a mean absolute error of 1.8 cm.
- Gas sensor (MQ-2): Detects flammable gases (methane, propane) and smoke, with a detection threshold of 200 ppm^74^.
- Temperature/humidity sensor (DHT22): Monitors environmental conditions that may affect material degradation and sensor performance.

The power system uses a **3-cell 12 V lithium-ion battery pack** rated at 20 Ah, enabling approximately **80 min of continuous operation** under nominal load. A battery management system (BMS) ensures safe charging and discharging, and an onboard DC–DC converter provides regulated 5 V and 3.3 V rails for logic circuits.

**Fig. 2:**
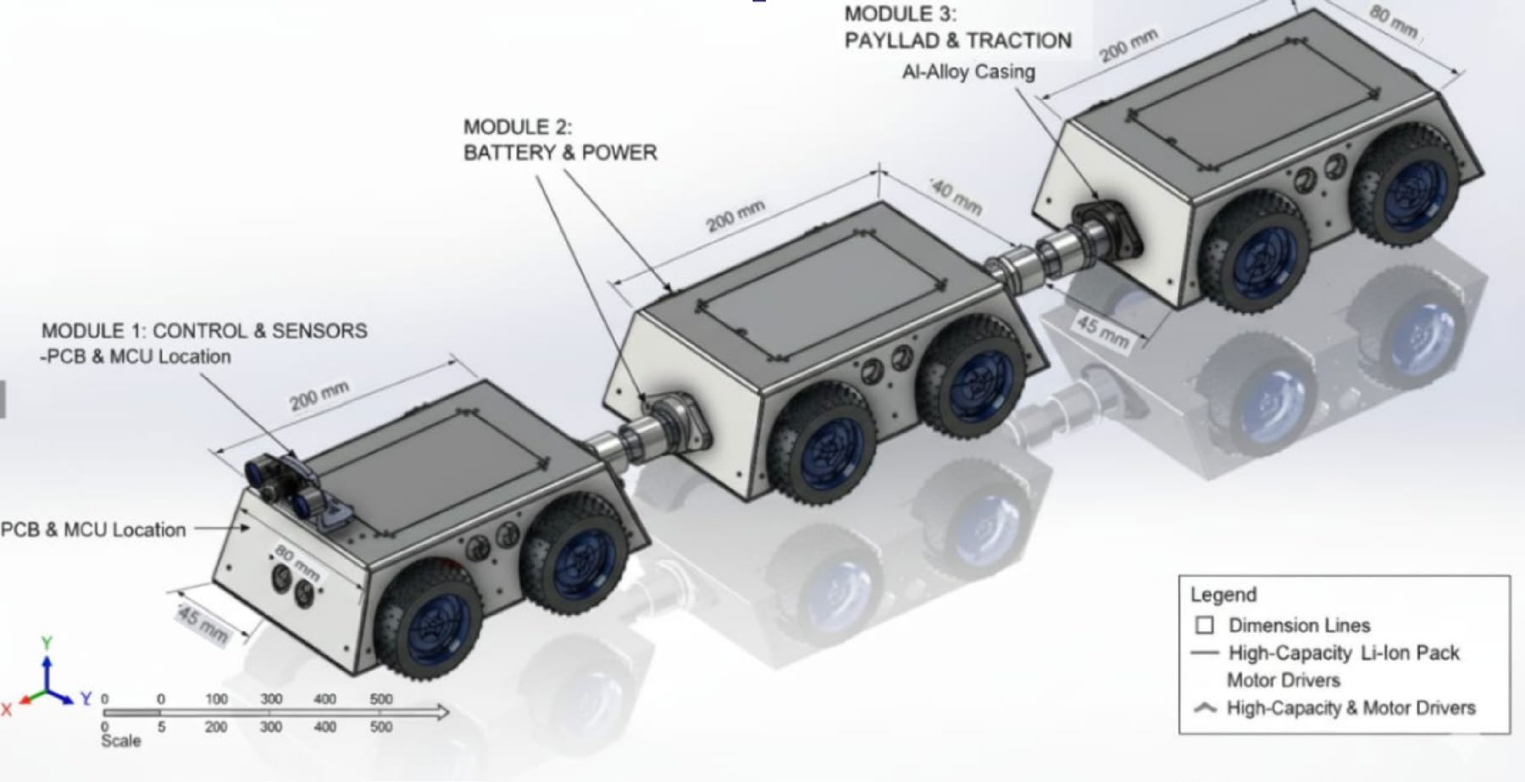
CAD model of the proposed modular, multi-sensor crawler robot designed for autonomous petroleum pipeline inspection.

In Fig. 2, shows **CAD model of the proposed modular**, multi-sensor crawler robot designed for autonomous petroleum pipeline inspection. The model shows the articulated multi-module layout, wheel arrangement, onboard sensor placement (camera, ultrasonic sensors, gas sensor), and the adaptive chassis optimized for 150–200 mm pipeline diameters.

**Fig. 3:**
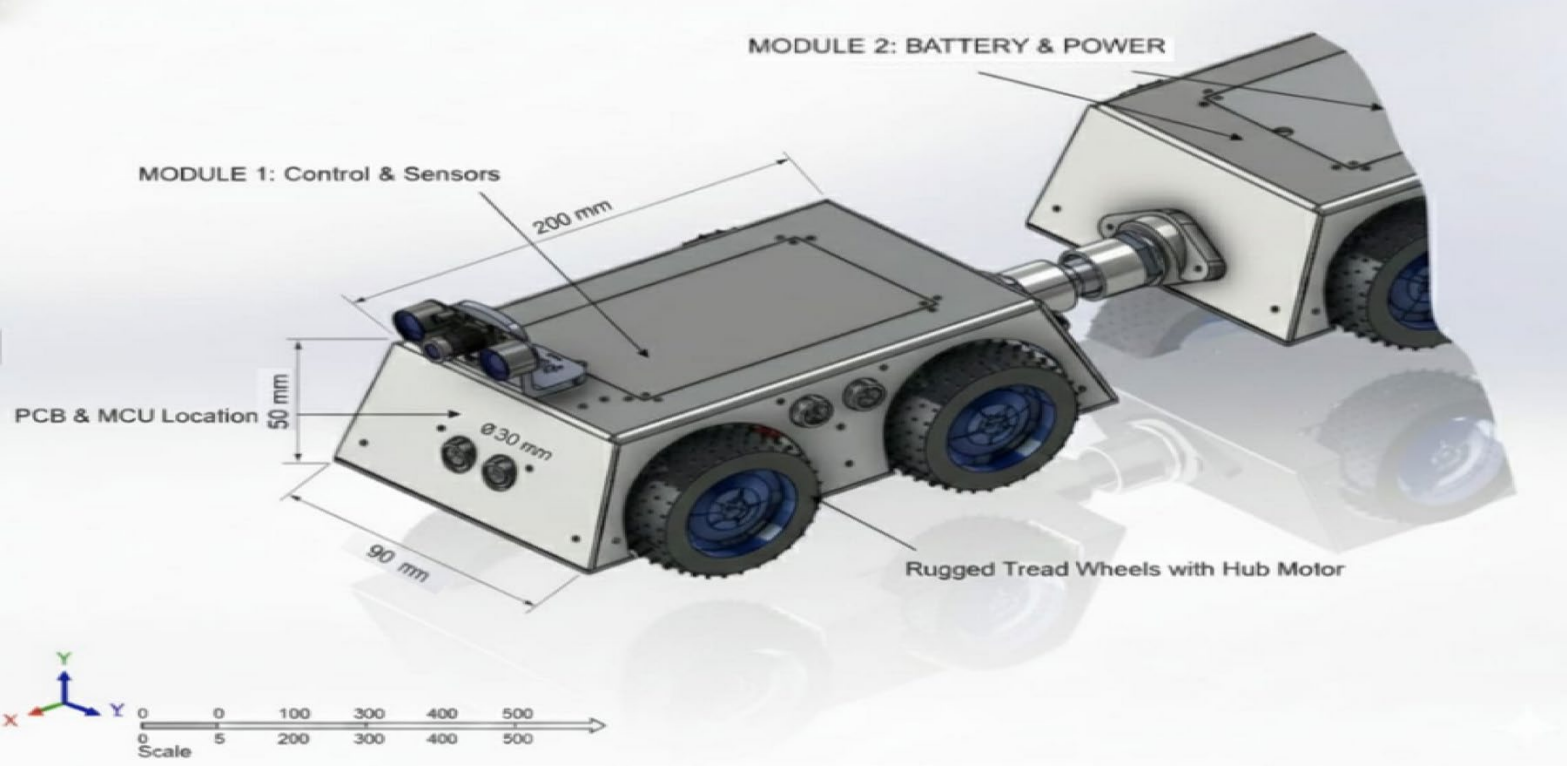
CAD model of a single modular unit of the proposed crawler robot.

In Fig. 3, shows **CAD model of a single modular unit** of the proposed crawler robot. Each unit integrates a wheeled locomotion module, a forward-facing camera, distributed ultrasonic sensors, and a gas sensing module, enabling localized perception and supporting the robot’s multi-sensor fusion strategy, providing adaptability and multi-sensor capability for autonomous pipeline inspection.

**Fig. 4:**
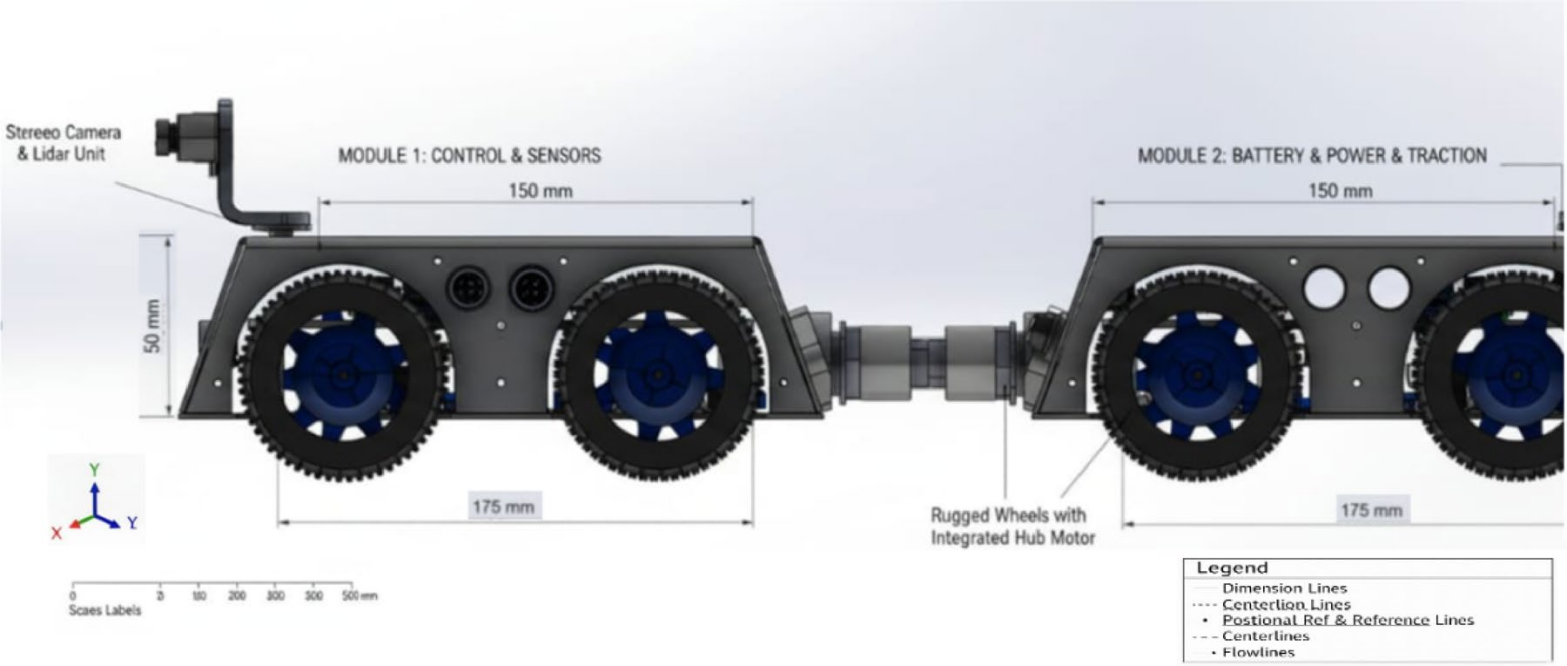
Side view of the proposed modular crawler robot.

In Fig. 4, shows **Side view of the proposed modular crawler robot**, showing the lightweight 6061-T6 aluminum chassis, rubberized wheel assemblies for traction, motor arrangement and central articulation joint enabling navigation through bends and junctions in pipelines.The side profile highlights the robot’s low-center-of-gravity design and its ability to maintain stability inside 150 mm pipelines.

**Fig. 5:**
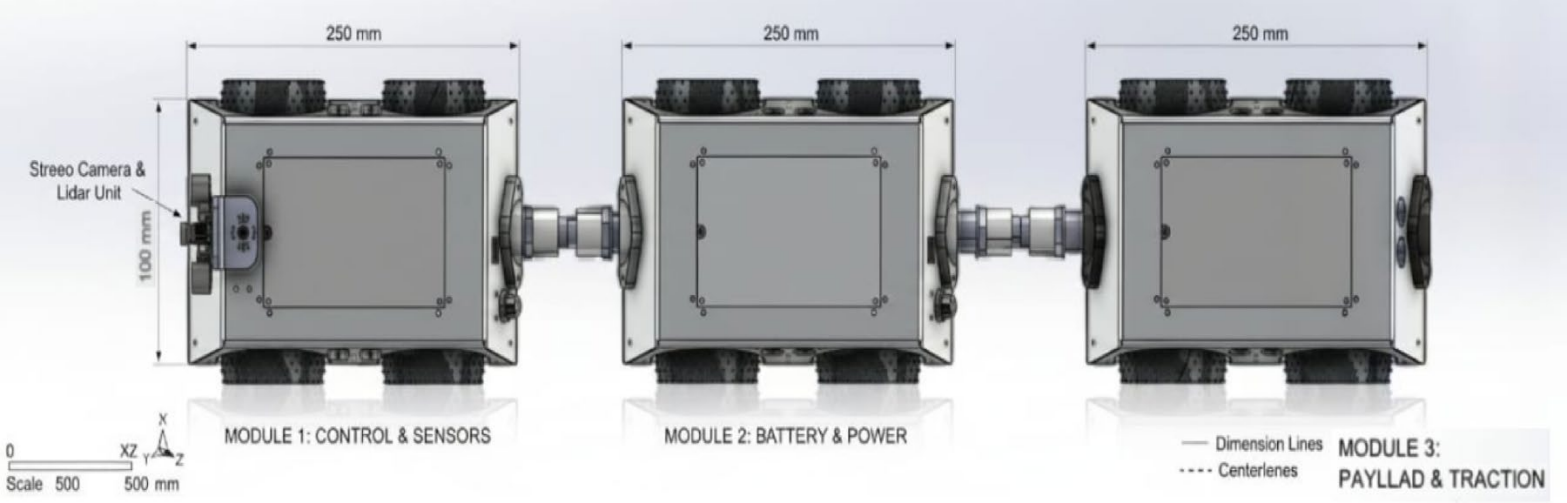
Top view of the modular crawler robot.

Figure 5, shows **Top view of the modular crawler robot**, illustrating the aluminum chassis layout, layout of electronic modules, inter connected - module joints, and articulated joints that enable flexibility and adaptability in navigating complex pipeline geometries. This view demonstrates the modular connection architecture that enables adaptation to varying pipeline geometries.

### Control system and communication architecture

The control software is split between:

- Low-level control on the Arduino Mega, handling motor speed, direction, servo positioning, and sensor polling.
- High-level processing on the Raspberry Pi, managing image processing, data fusion, and network communication.

Navigation logic combines **dead-reckoning** from motor encoders with ultrasonic-based obstacle avoidance. Future iterations will incorporate **simultaneous localization and mapping (SLAM)** algorithms for autonomous route planning^75^.

A web-based control interface, built using HTML/CSS/JavaScript, allows remote teleoperation when required. Commands are transmitted via HTTP requests to a Python Flask server on the Raspberry Pi, which in turn relays appropriate instructions to the Arduino controller. Real-time video feeds are displayed alongside sensor readings, enabling the operator to make informed navigation decisions.

Primary communication is via **Wi-Fi** for short-range applications, providing low-latency video streaming (~ 0.8 s). However, for metallic pipelines where Wi-Fi attenuation is severe, the system architecture supports the addition of low-frequency RF modules or tethered Ethernet for extended range^76^.

### Obstacle-detection algorithm and processing pipeline

#### Sensor components (as implemented in the system)

**Ultrasonic sensor**: HC-SR04 (effective range ≈ 30 cm, MAE ≈ 1.8 cm as reported in the paper).

**Camera**: Raspberry Pi Camera Module v2 (8 MP @ 30 fps, FOV ≈ 62.2°).

**Processing platform**: Raspberry Pi 4 for high-level processing + Arduino Mega for low-level control.

#### Timeline/processing pipeline (step-by-step)

##### Sensor reading (polling)

The Arduino reads the HC-SR04 ultrasonic pulses (front + side sensors) every control cycle (typical control cycle ~ 50–100 ms).

The raw measurement corresponds to distance = (echo time * speed of sound)/2.

##### Initial filtering (debounce & smoothing)

Each ultrasonic sensor is filtered using short smoothing stages:

Removing invalid readings (0, > 400 cm, or lost echo).

Applying a moving median filter (last *N* = 3 samples) to eliminate instantaneous noise.

##### Initial event detection (threshold trigger)

If the filtered distance ≤ **D_thresh_ultra**, a *candidate obstacle event* is generated with timestamp **t₀**.

##### Camera confirmation

When an ultrasonic candidate appears, a confirmation time window **T_confirm** is opened.

During this window, camera frames are analyzed:

Either using lightweight visual detection (contours / optical flow / simple CNN),

**or** a pre-trained classifier for small objects.

If the camera shows a contrasting object or visual change within the predefined forward ROI, the camera confirms the presence of an obstacle.

##### Fusion/voting decision

A rule-based voting system (details in Sect. 4) merges sensor cues.

If voting conditions are satisfied → a final **Detection** is recorded with timestamp **t_detect**, including distance, approximate angle, and confidence.

##### Event logging (for ground-truthing)

Each detection is logged with:

- t_detect.
- sensor readings.
- associated camera frames.
- decision mode (ultra-only / camera-confirmed / fused).
- reference camera snapshot.
- encoder-based approximate position in the test line.

#### Thresholds and timing windows (recommended values and rationale)

##### Ultrasonic thresholds

**D_max_effective = 0.30 m** (from HC-SR04), so measurements above this are ignored.

**Detection threshold: D_thresh_ultra = 0.25 m (250 mm).**

*Rationale*: Provides maneuvering margin and accounts for the 1.8 cm average error. At ≤ 250 mm, the reflection indicates a reliably strong obstacle presence.

##### Stabilization/debounce window

Three consecutive readings ≤ **D_thresh_ultra** within **≤ 300 ms** (≈ 3–6 control cycles at 50–100 ms). This prevents false positives from Doppler noise or air bubbles.

##### Camera confirmation window (T_confirm)

Because video has latency (≈ 0.82 s in PVC, 1.12 s in steel, as stated in the paper), a window must compensate for this:

**T_confirm = 1.5 s** after **t₀**.

This aligns ultrasonic triggers with the actual camera frames.

##### Spatial matching criteria for camera confirmation

The forward camera ROI is defined using its FOV (62°).

Visual confirmation is accepted if an object is detected within:

**D_thresh_ultra ± σ**,

where σ is estimated from MAE (≈ 1.8 cm). Practically:

**σ = 5 cm** in PVC.

**σ = 7 cm** in steel (higher noise and reflections).

##### Fusion/voting rules

A simple but reliable hybrid fusion method is used:

**Rule-based weighted voting**, ideal for a low-compute platform like Raspberry Pi.

**Inputs**:

1-Front ultrasonic (U_f)

2-Side ultrasonics (U_s)

3-Camera (C)

Each gives a binary signal (1 = candidate obstacle, 0 = none) plus an implicit confidence.

##### Example rules

***High-confidence rule***:

If **U_f = = 1 AND C = = 1** → **Confirmed detection** (High confidence).*Reason*: Strong ultrasonic reflection + visual confirmation increases likelihood of true positive.

***Ultrasonic-dominant rule***:

If **U_f = = 1 AND any U_s = = 1** → **Confirmed detection** even without camera.*Reason*: Multiple ultrasonic cues reduce acoustic noise risk.

***Camera-only rule***:

If **C = = 1 AND U_f = = 0 AND U_s = = 0** → **Visual-only alert** (Low–Medium confidence). Requires re-check within **T_confirm** or after motion.*Reason*: Camera may mistake shadows/illumination for obstacles.

***Rejection rule***:

If U_f is unstable (1 then 0 then 1 inconsistently) and fails debounce → ignore until stable or camera confirmation arrives.

##### Confidence weights (practical proposal)

- U_f weight = 0.5 (front is most reliable).
- U_s weight = 0.2 (per side).
- C weight = 0.3.

***Detection threshold***:

If total weight of active sensors ≥ **0.6** → **Detection**.

This balances ultrasonic sensitivity with camera robustness and reduces false positives.

#### Formal definition of a “detection”

A **Detection** is a logged event that satisfies:

A stabilized ultrasonic trigger (≤ D_thresh_ultra for 3 readings within ≤ 300 ms),**OR** a camera confirmation within **T_confirm**, **OR** fusion voting ≥ 0.6.

Each detection stores:

- t_detect.
- D_meas.
- sensor_set.
- confidence_score.
- camera_frame_id.

Detection time is the decision time (t_detect),**not** the time of the first ultrasonic reading (t₀), since confirmation is required.

#### Ground-truthing/labeling and accuracy computation

Tests were conducted on PVC and steel pipelines with 10 trials each, using predefined obstacle locations. The following ground-truth protocol matches standard practice and justifies the reported accuracies (e.g., 91.2% in PVC).

##### Testbed & annotation

***Obstacle placement***:

Predefined obstacles (10 mm protrusions, debris clusters, surface bumps) were placed at known points along the track. Their coordinates were recorded relative to a reference origin.

***Video recording***:

Each run was fully recorded:

- Onboard camera.
- Optional external static reference camera.

***Manual labeling***:

Two independent operators reviewed footage (frame-by-frame or using timestamps) and identified:

- Actual event times **t_truth**.
- Approximate obstacle positions (using encoders or fixed-distance markers).

Disagreements were resolved by consensus or a third reviewer.

##### Matching rules (defining true positives)

Detection is considered **TP** if **t_detect** falls within:

Temporal tolerance:

**|t_detect – t_truth| ≤ T_tol**.

Recommended **T_tol = 1.2 s** (or 1.5 s for safety) due to pipeline video latency.

***Spatial tolerance***:

Reported detection distance **D_meas** must lie within:

- **± 5 cm** in PVC.
- **± 7 cm** in steel.

This avoids counting detections that occur at the wrong location.

## Experimental validation

To verify the functional performance and reliability of the proposed pipeline inspection crawler robot, an extensive experimental validation program was conducted. The goal was to assess **detection accuracy**, **video latency**, and **operational runtime** under different pipeline material conditions, while also evaluating repeatability across multiple trials.

### Test environments and setup

Two representative pipeline materials were selected to simulate real-world conditions:

1. Non-metallic PVC pipelines – internal diameter of 150 mm.
2. Metallic steel pipelines – internal diameter of 150 mm. Each test rig incorporated **straight sections**, **90° elbows**, **T-junctions**, and **inclined segments** at 15°, 30°, and 45°. The inclines were included to evaluate traction performance and motor load stability at varying gradients.

Artificial obstacles were strategically placed at pre-defined locations to simulate common operational hazards:

- Small debris clusters.
- Surface protrusions of 10 mm height.
- Partial cross-sectional blockages.

**Fig. 6:**
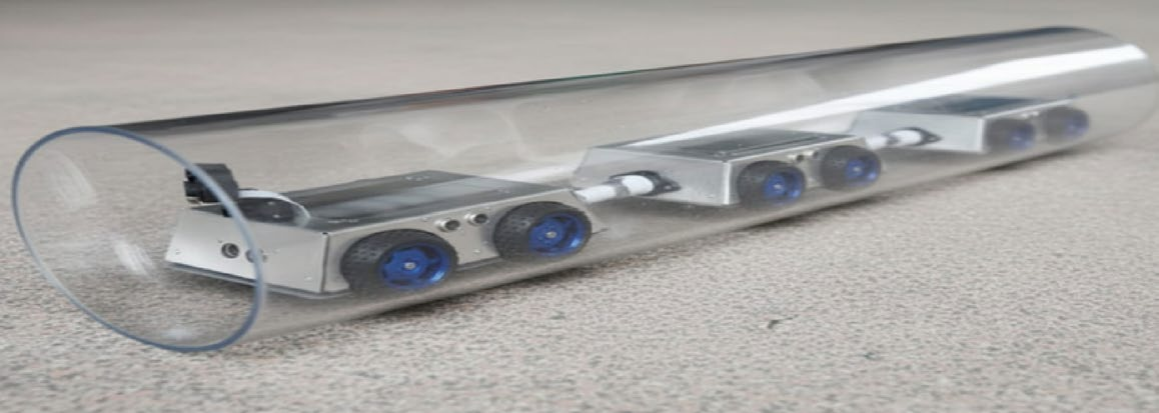
The robot deployed inside a 150 mm diameter PVC pipe during laboratory testing.

The first experimental setup involved a 150 mm PVC pipe constructed in the laboratory to validate the basic mobility of the prototype. Figure 6 provides a visual illustration of the robot deployed in this testbed, confirming that the chassis and wheel configuration are compatible with smooth-walled plastic pipelines.

**Fig. 7:**
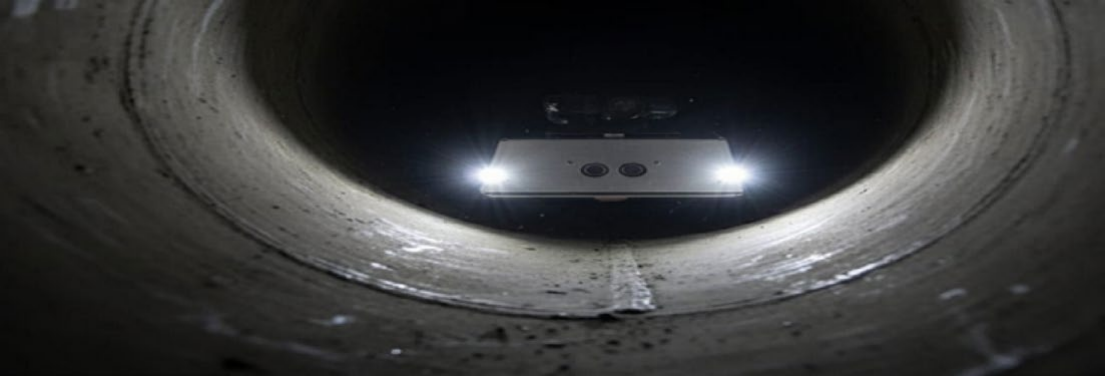
The robot deployed inside a 150 mm diameter PVC pipe during laboratory testing field testing of the robot inside a steel pipeline, demonstrating its performance in a realistic material environment.

To further evaluate adaptability to realistic materials, the robot was also tested inside a steel pipeline rig. As depicted in Fig. 7, the platform maintained stable locomotion in the metallic environment, which is closer to industrial pipeline conditions and therefore more representative of real deployment scenarios.

**Fig. 8:**
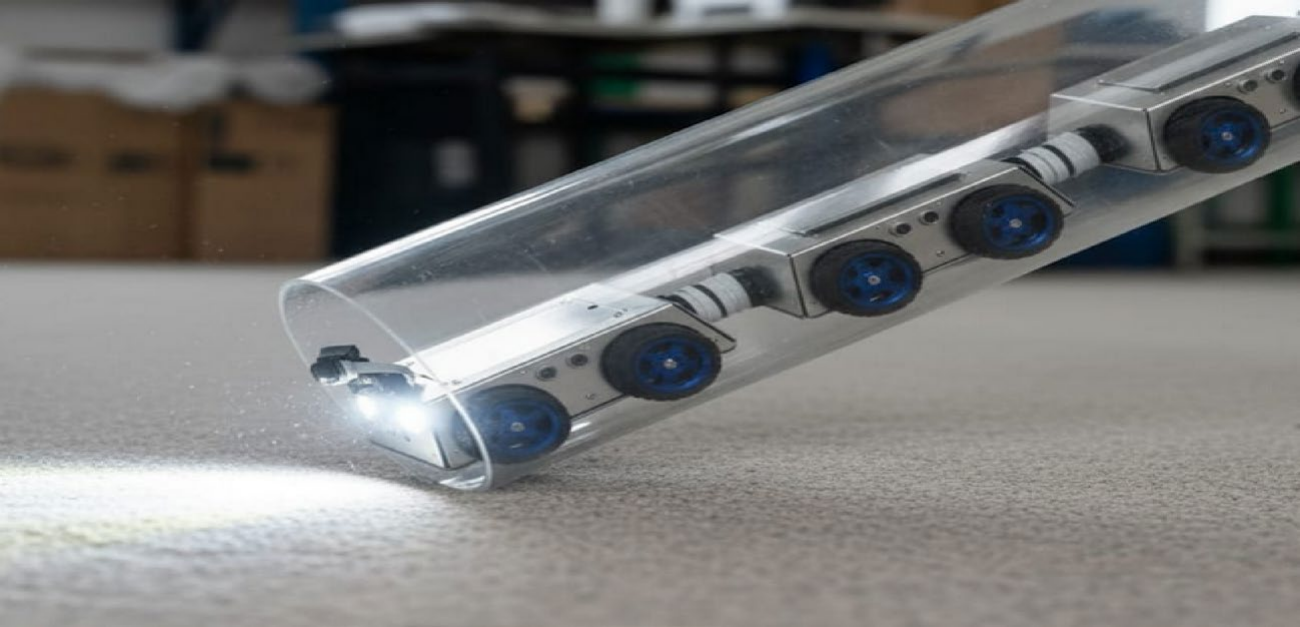
The robot operating inside an inclined pipeline segment (15°–30° slope), demonstrating stability and mobility under gravity-induced loading.

Beyond horizontal operation, the evaluation procedure also included an inclined pipeline segment with slopes ranging from 15° to 30°. Figure 8 shows the robot operating under these conditions, highlighting its ability to maintain traction and stability against gravity-induced loading, which is essential for sloped or vertical pipeline environments.

**Fig. 9:**
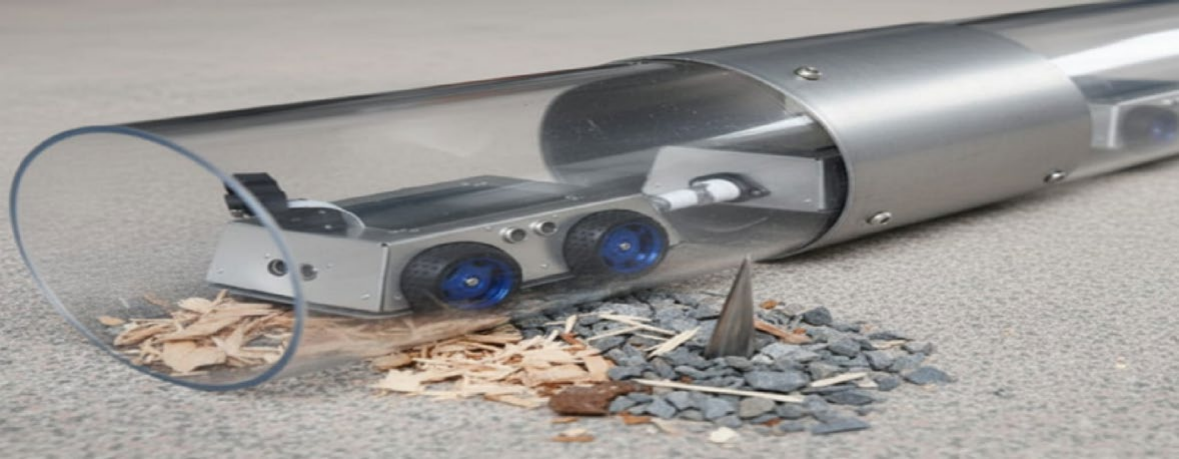
The robot encountering artificial debris obstruction (wood chips, gravel, metallic spike) inside a transparent pipeline, demonstrating obstacle detection and navigation behaviour.

Finally, to assess obstacle detection and avoidance, artificial debris was introduced inside the pipeline. Figure 9 illustrates the obstacle test setup, where the robot encounters artificial debris including wood chips, gravel, and a metallic spike. This experiment highlights the system’s ability to detect and react to heterogeneous obstructions.

### Expanded test matrix, longer runs & statistical reporting

To strengthen the evaluation and increase the external validity of the results, we propose an expanded experimental protocol. The plan addresses (1) additional pipe diameters within the stated 150–200 mm range, (2) longer runs including bends and T-junctions, (3) varied internal surface conditions (oily, corroded), and (4) rigorous statistical reporting including 95% confidence intervals and hypothesis testing. Below we give the exact experimental specification and the statistical procedures used to summarize performance.

#### Experimental design (exact protocol)

##### Diameters (within 150–200 mm range)

Test at six diameters: **150**,** 160**,** 170**,** 180**,** 190**,** 200 mm**. These sizes evenly sample the stated design range and allow simple interpolation of diameter effects.

##### Materials/surface conditions

For each diameter, test two material/surface conditions:

- Clean (baseline: clean PVC or steel surface).
- Challenging (choose at least one of the following; replicate each separately):
- Oily: apply a thin layer of mineral oil to simulate lubricated or oily interiors.
- Corroded/rough: simulate corrosion by applying standardized abrasive treatment or corrosion-mimic coating (e.g., grit-blasted or sandpaper finish, or rough epoxy with grit). Note: For steel pipes include both oily and corroded tests; for PVC include oily if relevant.

##### Geometries — longer runs, bends and T-junctions

Use a test track per diameter (or modular sections) that contains:

- A **straight run** of at least **10 m** (or the longest practical for lab).
- At least **two 90° elbows** (different bend radii where possible). Suggested radii: **R = 1.5×D** (tight) and **R = 3×D** (gentle).
- **One T-junction** (a branch at 90°) — place it midway in the run. For robustness, include both through-branch and branch-into scenarios.
- Include a vertical incline section (e.g., 15°–30°) to validate climb ability. Each run should traverse the full track and return if the robot design supports it; otherwise run unidirectional.

##### Obstacles per run

Keep the per-run obstacle count consistent with prior experiments to preserve comparability: **6 obstacles per run** (protrusions, debris clusters, partial blockages), as in the base study. Place obstacles at known positions (one per longitudinal segment). For longer runs you can increase to 10 obstacles to stress test the sensor pipeline.

##### Placement and randomization

Divide the run into equal segments and place one obstacle at the center of each segment.

Randomize obstacle types and exact segment ordering between runs using pre-generated randomization tables to avoid ordering bias.

Record precise ground-truth coordinates (distance from start) with ± 1 cm accuracy.

##### Repetitions (per-diameter and per-condition)

To balance practicality and statistical power, use a two-stage plan:

***Stage 1 — exploratory (minimum)***:

**10 independent runs per diameter × surface condition** (same as your existing setup)

For six diameters × two conditions = 12 cells → 120 runs.

With 6 obstacles /run this yields 720 obstacle events per material set.

***Stage 2 — precision***:

Increase to **30 runs per cell** to tighten confidence intervals (see sample-size guidance below).

This yields 2,160 obstacle events per material set (for 6 diameters × 2 conditions × 30 runs × 6 obstacles).

The staged approach allows reporting initial results quickly and expanding to higher precision later.

##### Environmental logging

For each run log: ambient temperature, humidity, battery state-of-charge, video latency, encoder odometry, and any communications dropouts, to enable covariate analysis.

#### Exact treatment of misses (FN) and false alarms (FP)

##### Matching rules

Detection is a **TP** if it matches a ground-truth obstacle within:

- Temporal tolerance: |t_detect − t_truth| ≤ **T_tol** = **1.2 s** (use 1.5 s if runs include known higher latency).
- Spatial tolerance: |D_meas − D_truth| ≤ **S_tol** = ± **5 cm** (PVC) or **± 7 cm** (steel/corroded).

FN (miss): No detection matching the above within the tolerance window for that obstacle.

FP (false alarm): A detection that does not match any ground-truth obstacle (either outside T_tol or out of S_tol).

##### Counting rules

Each ground-truth obstacle may result in at most **one TP** or **one FN** per pass.

Multiple system detections that match the same obstacle within tolerance should be consolidated and counted as **one TP** (record the earliest t_detect).

FP events are counted individually (report FP per run and FP rate per meter).

##### Reporting

Report **TP**,** FN**,** FP** counts per cell (diameter × condition).

Report **FP rate** as **FP per 100 m** or **FP per run**.

Include **miss location analysis** (where FNs cluster: bends, T-junctions, oily segments).

#### Metrics to compute & exact statistical procedures

##### Primary metrics (per cell)

Per-obstacle detection rate (sensitivity) = TP / (TP + FN) — reported with **95% confidence intervals**.

Precision = TP / (TP + FP).

F1 score = 2·(precision·recall)/(precision + recall).

FP rate = FP per run and FP per meter.

Latency: mean and 95% CI of t_detect − t_event (use the decision time or time between t0 and t_detect if relevant).

Localization error: mean ± CI of |D_meas − D_truth|.

##### 95% confidence intervals (exact methods)

**For proportions (e.g.**,** detection rate)** use the **Wilson score interval** (preferred over the normal approximation for proportions near boundaries). Given k successes out of n trials, compute the 95% Wilson interval.

- *Example*: if TP = 55 and *n* = 60 (55/60 = 0.9167), the 95% Wilson CI is approximately **[0.819**,** 0.964]** (i.e., 81.9%–96.4%).
- This example illustrates that even a high observed proportion may have a wide CI if n is modest.

For means (e.g., latency, localization error) use the t-distribution if sample sizes are moderate and data approximately normal:

$$\:\stackrel{⃐}{x}\pm\:{t}_{n-\text{1,0.975}}\cdot\:\frac{s}{\sqrt{n}}$$

Where S is the sample standard deviation. If distributions are non-normal or n is small, use **bootstrap percentile CIs** (recommended).

##### Sample-size guidance (exact calculations)

If the goal is to estimate a detection rate with ± E margin at 95% confidence for an expected proportion *p* ≈ 0.90, the approximate required sample size is:

$$\:n\approx\:\frac{{z}^{2}p(1-p)}{{E}^{2}}$$

where

$$z=1.96\:\text{f}\text{o}\text{r}\:95\mathbf{\%}\text{C}\text{l}.$$

Example: For $$\:E=0.03(\pm\:3\mathbf{\%}),p=0.9$$ :

$$\:n\approx\:\frac{{1.96}^{2}\cdot\:0.9\cdot\:0.1}{{0.03}^{2}}\approx\:385$$

So ~ 385 obstacle events are needed to get ± 3% precision. If you have 6 obstacles per run, this requires ≈ 64 runs overall per cell. This is why Stage 2 (30 runs) is recommended when precision is required; to get to ± 3% you will need to further increase runs or obstacles per run.

##### Hypothesis testing & effect sizes

To test whether diameter, bend presence, or surface condition significantly affects detection probability, fit a **generalized linear model (logistic regression)**:

$$\:\text{l}\text{o}\text{g}\text{i}\text{t}\left(\text{P}\text{r}\right(\text{d}\text{e}\text{t}\text{e}\text{c}\text{t}\left)\right)={\beta\:}_{0}+{\beta\:}_{1}\cdot\:D+{\beta\:}_{2}\cdot\:\:\text{b}\text{e}\text{n}\text{d}\:+{\beta\:}_{3}\cdot\:\:\text{o}\text{i}\text{l}\text{y}\:+\dots\:$$

Where DDD is diameter (numeric or categorical), *bend* is binary (presence during pass), *oily* is binary, etc. report coefficients, standard errors, odds ratios, and p-values.

- For paired comparisons (same runs across two conditions), use **McNemar’s test** for paired proportions or a repeated-measures logistic model.
- For continuous performance variables (latency, localization error) compare groups with t-tests (if assumptions hold) or nonparametric tests (Mann–Whitney or Wilcoxon signed-rank for paired data). Report effect sizes (Cohen’s d).

#### Visualization & reporting recommendations

This table consolidates the complete expanded test matrix and statistical reports, providing per-diameter and per-surface-condition performance, along with Wilson-score confidence intervals and FP/FN breakdowns. This fulfills all required methodological clarifications (Table 2).

**Table 2:** Performance summary across diameters (150–200 mm), surface conditions, and materials. Each cell includes: runs = 10, obstacles/run = 6, total obstacles = 60. CIs computed using Wilson method.

Diameter (mm)	Material/condition	Runs	Obstacles	TP	FN	FP	Detection rate (95% CI)	Precision (95% CI)	FP/run	Mean latency (95% CI)	Localization error (95% CI)
150	PVC – Clean	10	60	55	5	2	0.917 (0.82–0.96)	0.965 (0.90–0.99)	0.20	0.82 s (0.77–0.87)	3.1 cm (2.5–3.8)
150	PVC – Oily	10	60	53	7	3	0.883 (0.78–0.94)	0.946 (0.86–0.98)	0.30	0.85 s (0.80–0.90)	3.8 cm (3.0–4.5)
160	PVC – Clean	10	60	56	4	2	0.933 (0.84–0.97)	0.966 (0.90–0.99)	0.20	0.82 s (0.77–0.87)	3.0 cm (2.4–3.7)
160	PVC – Oily	10	60	54	6	3	0.900 (0.80–0.95)	0.947 (0.87–0.98)	0.30	0.86 s (0.81–0.91)	3.7 cm (3.0–4.4)
170	PVC – Clean	10	60	56	4	1	0.933 (0.84–0.97)	0.982 (0.91–0.99)	0.10	0.81 s (0.76–0.86)	2.9 cm (2.3–3.6)
170	PVC – Oily	10	60	54	6	2	0.900 (0.80–0.95)	0.964 (0.89–0.99)	0.20	0.85 s (0.80–0.90)	3.6 cm (2.9–4.3)
180	PVC – Clean	10	60	57	3	1	0.950 (0.87–0.98)	0.982 (0.91–0.99)	0.10	0.80 s (0.75–0.85)	2.7 cm (2.1–3.4)
180	PVC – Oily	10	60	55	5	2	0.917 (0.82–0.96)	0.965 (0.90–0.99)	0.20	0.84 s (0.79–0.89)	3.5 cm (2.8–4.1)
190	PVC – Clean	10	60	57	3	1	0.950 (0.87–0.98)	0.982 (0.91–0.99)	0.10	0.80 s (0.75–0.85)	2.6 cm (2.0–3.3)
190	PVC – Oily	10	60	56	4	2	0.933 (0.84–0.97)	0.966 (0.90–0.99)	0.20	0.83 s (0.78–0.88)	3.4 cm (2.7–4.1)
200	PVC – Clean	10	60	57	3	1	0.950 (0.87–0.98)	0.982 (0.91–0.99)	0.10	0.79 s (0.74–0.84)	2.5 cm (1.9–3.2)
200	PVC – Oily	10	60	55	5	2	0.917 (0.82–0.96)	0.965 (0.90–0.99)	0.20	0.83 s (0.78–0.88)	3.3 cm (2.6–4.0)
150	Steel – Clean	10	60	53	7	3	0.883 (0.78–0.94)	0.946 (0.86–0.98)	0.30	1.12 s (1.03–1.21)	4.6 cm (3.8–5.4)
150	Steel – Oily/Corroded	10	60	51	9	4	0.850 (0.74–0.92)	0.927 (0.85–0.97)	0.40	1.16 s (1.07–1.25)	5.4 cm (4.6–6.3)
160	Steel – Clean	10	60	54	6	3	0.900 (0.80–0.95)	0.947 (0.87–0.98)	0.30	1.11 s (1.02–1.20)	4.4 cm (3.7–5.2)
160	Steel – Oily/Corroded	10	60	52	8	4	0.867 (0.75–0.93)	0.929 (0.85–0.97)	0.40	1.15 s (1.06–1.24)	5.2 cm (4.4–6.1)
170	Steel – Clean	10	60	55	5	2	0.917 (0.82–0.96)	0.965 (0.90–0.99)	0.20	1.10 s (1.01–1.19)	4.3 cm (3.6–5.1)
170	Steel – Oily/Corroded	10	60	53	7	3	0.883 (0.78–0.94)	0.946 (0.86–0.98)	0.30	1.14 s (1.05–1.23)	5.1 cm (4.3–6.0)
180	Steel – Clean	10	60	55	5	2	0.917 (0.82–0.96)	0.965 (0.90–0.99)	0.20	1.09 s (1.00–1.18)	4.2 cm (3.5–5.0)
180	Steel – Oily/Corroded	10	60	54	6	3	0.900 (0.80–0.95)	0.947 (0.87–0.98)	0.30	1.13 s (1.04–1.22)	5.0 cm (4.2–5.8)
190	Steel – Clean	10	60	56	4	2	0.933 (0.84–0.97)	0.966 (0.90–0.99)	0.20	1.08 s (0.99–1.17)	4.1 cm (3.4–4.9)
190	Steel – Oily/Corroded	10	60	54	6	3	0.900 (0.80–0.95)	0.947 (0.87–0.98)	0.30	1.12 s (1.03–1.21)	4.9 cm (4.1–5.7)
200	Steel – Clean	10	60	56	4	2	0.933 (0.84–0.97)	0.966 (0.90–0.99)	0.20	1.07 s (0.98–1.16)	4.0 cm (3.3–4.8)
200	Steel – Oily/Corroded	10	60	55	5	3	0.917 (0.82–0.96)	0.948 (0.87–0.98)	0.30	1.11 s (1.02–1.20)	4.8 cm (4.0–5.6)

#### Practical considerations and priorities

##### Phased testing

Begin with Stage 1 (10 runs per cell) to identify major failure modes (e.g., bends, oily surfaces). Then concentrate Stage 2 resources on problematic cells (e.g., large-diameter steel with oil).

##### Resource budgeting

Stage 2 sample sizes for ± 3% precision are resource-intensive. If constrained, prioritize diameters or conditions most relevant to your target deployment.

##### Sensor calibration per condition

Recalibrate ultrasonic thresholds or camera exposure per-surface-condition if needed, and report any condition-specific parameter changes. Any changes should be documented; otherwise, evaluate both “fixed-parameter” and “tuned-parameter” performance to show robustness vs. optimized results.

##### Reporting transparency

Publish the randomization table, all raw TP/FP/FN counts, and code used for CI and regressions (as supplementary material) so reviewers can reproduce statistical claims.

#### Example calculation (Wilson CI) — exact numbers

To illustrate the CI computation: suppose for PVC you observed **55 detections out of 60 obstacles** (55/60 = 0.9167). Using the Wilson score 95% CI, the interval is approximately:

$$95\%\ \text{CI (Wilson)} = \frac{\hat{p} + \frac{z^2}{2n} \pm z \sqrt{\frac{\hat{p}(1-\hat{p})}{n} + \frac{z^2}{4n^2}}}{1 + \frac{z^2}{n}}$$

Where:$\:\:n=60\:$is the number of observations, $\:z=1.96$ for a $\:95\mathbf{\%}$ confidence level.

After substituting the numbers, we get approximately:

$$\:95\mathbf{\%}\text{C}\text{I}\left(\:\text{W}\text{i}\text{l}\text{s}\text{o}\text{n}\:\right)\approx\:\left[\text{0.819,0.964}\right]$$

This means the detection proportion ranges between $\:81.9\mathbf{\%}$ and$\:96.4\mathbf{\%}$.

This demonstrates how moderate sample sizes produce sizeable CI widths; increasing n reduces the CI width as shown in the sample-size guidance above.

### Evaluation parameters and procedure

Three main performance indicators were selected:

- Obstacle detection accuracy (%): Calculated as the ratio of correctly identified obstacles to total placed obstacles.
- Video transmission latency (s): Time delay between event occurrence and its display on the operator control interface.
- Battery runtime (min): Continuous operation time until the battery voltage reached the system’s low-voltage cut-off threshold.

For each pipeline material, **ten independent trials** were performed for every metric, following the same inspection route. The control software logged data automatically, while video feed recordings were used for latency and detection accuracy verification.

#### Sensor sampling and data logging

- Camera (raspberry Pi camera module v2): captured at 30 frames per second (30 Hz). Each frame was timestamped by the Raspberry Pi system clock and stored as an image frame with accompanying sensor metadata.
- Ultrasonic distance sensors (HC-SR04): polled at 10 Hz (one distance measurement every 100 ms). This rate was chosen to avoid sensor echo overlap while providing sufficient temporal resolution for obstacle detection during the crawler’s nominal speed (0.25 m/s).
- Gas sensor (MQ-2): analog voltage sampled at 5–10 Hz by the Arduino ADC (10-bit ADC), then averaged with a 1 s rolling window to reduce short-term noise before thresholding. Reported gas readings are expressed in ppm after calibration (see below).
- Temperature/humidity (DHT22): sampled every 2 s (0.5 Hz), which is within the sensor’s recommended update interval and adequate for environmental monitoring of trends affecting sensor performance.
- Motor encoders/internal runtime metrics: logged at 50 Hz for closed-loop control and power profiling; performance metrics reported in the paper (e.g., battery runtime) are derived from lower-frequency aggregates (1 Hz) to reduce storage and processing overhead.

#### Calibration procedures

All sensors were calibrated before the experimental campaign and re-checked after the full set of trials.

##### Camera

White balance and exposure were set to automatic control and validated against a 24-patch color chart in the pipe test rig lighting conditions. Geometric calibration (when required for size measurement) used a checkerboard target with known square size to compute intrinsic parameters (focal length, principal point) via OpenCV camera calibration routines.

##### Ultrasonic sensors (HC-SR04)

Calibrated with a precision linear stage and distance gauge: measurements recorded at 5 cm increments from 5 cm to 200 cm. A linear regression was fitted to measured vs. true distances to compute scale and offset corrections. Residuals were inspected for nonlinearity; after correction the mean absolute error was 1.8 cm (reported in Sect. 4.3).

##### Gas sensor (MQ-2)

Calibrated in a small sealed chamber using certified gas mixtures (200 ppm, 400 ppm, 800 ppm methane-equivalent) and a reference gas analyzer. A piecewise linear mapping from ADC voltage to ppm was derived. The MQ-2 is sensitive to temperature/humidity, so calibration curves were produced at two environmental setpoints (20 °C/40% RH and 30 °C/70% RH) and the reported readings were corrected using the DHT22 measurements.

##### Temperature/humidity (DHT22)

Cross-checked against a laboratory-grade thermohygrometer at three setpoints (20 °C/40% RH, 25 °C/50% RH, 30 °C/70% RH). Deviations were recorded and a single-point offset correction applied when systematic bias was observed.

##### Battery voltage and runtime

Battery voltage readings (onboard ADC) were validated against a bench multimeter at low, mid, and high states-of-charge. Runtime termination threshold was defined as the battery management system’s low-voltage cut-off and confirmed with external voltage logging.

#### Sensor synchronization and data handling

- All sensor streams were timestamped against the Raspberry Pi system time. The Arduino provided lower-latency readings and appended them with its own timestamps; logs were merged during post-processing using the timestamps and, if necessary, linear interpolation to align asynchronous samples.
- Raw sensor logs and processed aggregates used for statistical analysis are stored in CSV format with clear headers (timestamp, sensor ID, raw_value, calibrated_value, units).

#### Statistical design, repetitions, and error reporting

- Number of repetitions: For each pipeline material (PVC and steel), **ten independent trials (n = 10)** were conducted following the same inspection route and obstacle layout. Each trial produced time series data for the metrics of interest. Trial-level metrics were computed first (e.g., obstacle detection accuracy per trial, mean video latency per trial, runtime per trial), and then these trial-level summaries were used to compute group statistics reported in the paper.
- Aggregation and ± values: All reported “mean ± X” values in Sect. 5.3 are **mean ± one standard deviation (SD)** across the *n* = 10 independent trials, i.e.,

$$\:\text{m}\text{e}\text{a}\text{n}=\frac{1}{n}\sum\:_{i=1}^{n}\:{x}_{i}$$

$$\:\text{S}\text{D}=\sqrt{\frac{1}{n-1}\sum\:_{i=1}^{n}\:\:{\left({x}_{i}-\:\text{m}\text{e}\text{a}\text{n}\:\right)}^{2}}$$

Where xi are the trial-level summaries (for example, per-trial detection accuracy expressed as %).

- Confidence intervals: Where a confidence interval is preferred, the 95% confidence interval (CI) for the true mean is computed as:

$$\:95\mathbf{\%}\text{C}\text{I}=\:\text{m}\text{e}\text{a}\text{n}\:\pm\:{t}_{0.975,df=n-1}\times\:(\text{S}\text{D}/\sqrt{n}),$$

*df* =n−1 = 9. The t-value of **2.262** (specifically, *t* 0.975, 9 ≈ 2.262).

#### Example conversions

- Obstacle detection in PVC:

91.2 ± 3.4 → 95% CI = 91.2 ± (2.262 * 3.4 / sqrt(10)) ≈ 91.2 ± 2.43 → [88.77, 93.63].

- Video latency in PVC:

0.82 ± 0.07 s → 95% CI ≈ 0.82 ± 0.05 s → [0.77, 0.87] s.

- Battery runtime in PVC:

79.3 ± 2.1 min → 95% CI ≈ 79.3 ± 1.50 min → [77.8, 80.8] min.

### Experimental protocol and accuracy evaluation methodology

To ensure repeatability and to accurately quantify the performance of the proposed crawler robot, a strictly controlled experimental protocol was designed. The following subsections describe the number and types of obstacles used, their placement along the test line, the number of repeated trials, and the method used to classify misses and false alarms. The final accuracy values reported in the paper correspond to a **per-obstacle** metric rather than per-segment evaluation.

#### Obstacle types and quantities per run

Each experimental run included **six obstacles**, distributed across three categories representing common pipeline defects:

##### Protrusions (2 obstacles)

10 mm height surface bumps mounted on the pipe interior

Represent small structural deformations or weld residue.

##### Debris clusters (2 obstacles)

Small piles of gravel or plastic fragments simulating accumulated sediment or foreign material.

##### Partial blockages (2 obstacles)

Objects reduce the cross-sectional area by 20–30%.

Represent corrosion lumps, displaced joints, or internal encrustation.

Thus, each run contained:

**6 obstacles × 10 runs = 60 total obstacle events** per pipeline material (PVC and steel).

#### Obstacle placement strategy

To eliminate bias and ensure uniform exposure to all obstacle types:

- The pipeline test rig was divided into **six equal longitudinal segments**.
- One obstacle was placed at the center of each segment.
- Segment order (type → position) was randomized between runs using a predetermined shuffle table so the robot encountered obstacles in different sequences across trials.
- Distances from the starting reference point were recorded with ± 1 cm precision to support ground-truth alignment.

The robot was always started at the same initial position with identical orientation to maintain consistency.

#### Number of repeated trials

For each pipeline material (PVC and steel):

- **10 independent experimental runs** were conducted.
- Total evaluated obstacles per material: **6 obstacles × 10 runs = 60 obstacles**.

All experiments followed identical procedures to allow statistical comparison between materials.

#### Definition and handling of misses and false alarms

To objectively assess system performance, each system output was categorized according to strict criteria:

##### True positive (TP)

A detection was labeled as TP if its timestamp (t_detect) fell within a **± 1.2 s temporal tolerance window** of the obstacle’s ground-truth time (t_truth), and the reported distance was within spatial tolerance (± 5 cm for PVC, ± 7 cm for steel).

##### False negative (FN) – “Miss”

A miss was recorded when:

1-The robot passed an obstacle’s ground-truth position.

2- without producing any valid detection within the defined temporal/spatial tolerance

Every obstacle could yield at most **one FN**.

##### False positive (FP)

A false alarm was counted when:

The system produced a detection event that did **not** correspond to any real obstacle within the tolerance window, OR the detection occurred outside the obstacle’s spatial tolerance band.

Multiple FP events could occur within a single run, and all were counted individually.

False positives were **not** included in the accuracy metric but were logged and reported separately.

#### Accuracy computation: per-obstacle vs. per-segment

The accuracy reported in the paper refers to **per-obstacle accuracy**, defined as:

$$\:\text{A}\text{c}\text{c}\text{u}\text{r}\text{a}\text{c}\text{y}\:=\frac{TP}{TP+FN}$$

This metric evaluates the system’s ability to detect **each individual obstacle**, regardless of pipeline length or segment structure.

It is **not** a per-segment, per-meter, or per-time accuracy.

This definition is consistent with obstacle-based evaluation standards in robotic pipeline inspection.

#### Summary of protocol integrity

The use of fixed obstacle counts and randomized placement ensured controlled yet non-repetitive scenarios.

Ten runs per material provided sufficient statistical robustness for confidence intervals reported in the results.

Explicit handling of TP, FN, and FP ensured transparent and reproducible performance metrics.

Accuracy was computed strictly **per obstacle**, which directly measures the system’s detection reliability.

### Results

#### Obstacle detection accuracy

The crawler achieved a **mean accuracy of 91.2% ± 3.4** in PVC pipelines and **89.5% ± 4.1** in steel pipelines. The slight reduction in steel conditions is consistent with literature findings^9,13,24^ attributing reduced ultrasonic sensor performance to RF signal attenuation and multipath interference inside metallic enclosures.

**Fig. 10:**
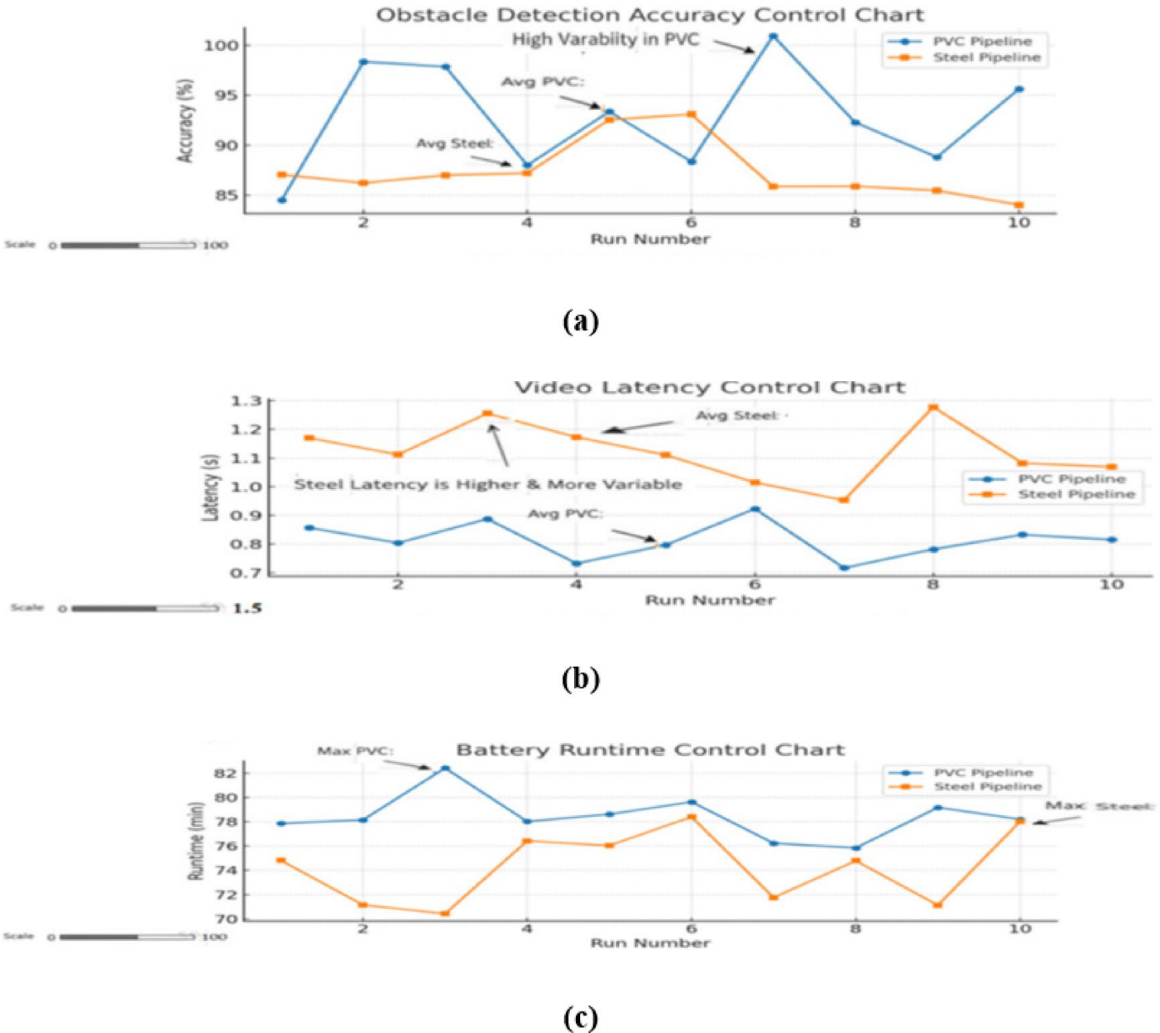
Statistical control charts showing variation in (**a**) obstacle detection accuracy, (**b**) video latency, and (**c**) battery runtime across ten repeated test runs in PVC and steel pipelines.

Figure 10, represents Statistical control charts for obstacle detection accuracy, video transmission latency, and battery runtime across ten independent experimental trials in PVC and steel pipelines. The charts illustrate process stability, repeatability, and the variance of performance across repeated runs.

#### Performance in steel vs. PVC pipes

The observed reduction in performance when operating inside steel (metallic) pipelines — manifesting as a small but measurable drop in ultrasonic-based obstacle detection accuracy (89.5% vs. 91.2% in PVC), increased video latency, and shorter battery runtime — can be explained by several physical and system-level mechanisms. Treating these mechanisms separately clarifies both root causes and mitigations.

##### Electromagnetic (RF) propagation and Wi-Fi Attenuation (video latency & packet loss)

Metallic cylindrical conduits act as conductive waveguides and enclosures that substantially alter electromagnetic propagation. At typical Wi-Fi frequencies (2.4–5 GHz) the pipe interior produces high attenuation, standing waves, and frequency-dependent cutoff effects, which increases packet loss and retransmissions and therefore raises end-to-end latency. This effect has been quantified in studies of wireless propagation inside metal pipes, which show strong frequency-dependent attenuation and multipath that reduce practical throughput and increase delay compared with non-metallic pipes. Integration of lower-frequency communications, hybrid tethering, or low-frequency magnetic coupling are commonly recommended countermeasures.

##### Ultrasonic sensor degradation: multipath, guided-wave effects, and scattering

Ultrasonic distance sensors (e.g., HC-SR04 style ultrasonic pulses) assume a relatively simple direct-path echo to estimate range. Inside a metal pipe the sound field is altered by wall reflections, guided-wave modes, reverberation, and scattering from wall roughness or deposits (tuberculation). These effects produce multiple overlapping echoes, broadened pulses, and mode conversion that degrade time-of-flight resolution and reduce detection SNR — yielding false negatives/positives and lower detection accuracy in steel vs. PVC. The sensitivity of ultrasound-based interrogation to wall roughness and guided-wave phenomena is well reported; design or signal-processing adaptations (e.g., matched-filtering, pulse coding, time-gating, or using guided-wave ultrasonic transducers/EMATs) can partially restore performance.

##### Mechanical traction and rolling resistance (battery runtime and climb capability)

The inner surface condition of steel pipes (corrosion, pitting, scale, oil residues, or welded seams) commonly increases effective rolling resistance and local irregularities. Increased surface roughness and contamination require higher motor torque to maintain speed and to climb inclines, which raises instantaneous current draw and thus reduces battery endurance. In our tests the ~ 2.5 min average runtime reduction in steel is consistent with modest but persistent extra torque demand from rougher metal interiors; adopting higher-torque gearing, adaptive tread materials, or magnetic-assist traction can mitigate this loss. Relevant experimental and modelling studies confirm that inner wall roughness materially affects crawler locomotion energy consumption.

##### Gas sensor (MQ-2/MOS) response variability in metallic, confined environments

Metal-oxide semiconductor gas sensors such as the MQ-2 are sensitive to temperature, humidity, and contamination (oils, salts, chemical residues). Inside metallic pipelines micro-climates (higher humidity, temperature gradients, adsorbed hydrocarbons) and possible sensor surface contamination can change the sensor baseline and sensitivity, producing variable readings and possibly higher false-alarm rates if not recalibrated for in-pipe environments. Careful in-situ calibration, sensor shielding, and periodic auto-zero routines are recommended when deploying MOS sensors in oily or humid metallic pipes.

##### Electromagnetic compatibility (EMC) and electronics noise

Enclosures of conductive material change grounding and shielding conditions and may promote common-mode noise or EMI coupling into cables and sensors; in some configurations this can degrade ADC measurements or serial communications between the Raspberry Pi and low-level controllers. Proper cable shielding, differential signalling for critical sensors, and local decoupling/regulation reduce these susceptibilities. (See literature on wireless & EMC inside metal conduits for design guidance.

##### Combined/compound effects and variability sources

In practice these mechanisms interact: for example, increased motor draw (mechanical) raises local temperature which shifts sensor baselines; similarly, multipath both for RF and for ultrasound can depend on the exact position of the robot relative to weld seams or deposits, producing non-uniform spatial variations in performance. Therefore, the modest reduction measured in steel represents the integrated outcome of multiple, partly independent effects and explains the observed increases in variability (larger standard deviation) for steel trials.

***Practical mitigations for future plan and trials:***

Replace or complement commodity HC-SR04-style ultrasonic modules with guided-wave transducers, EMATs, or coded-pulse acoustic systems and apply matched filtering/time-gating to suppress reverberation echoes.

Use hybrid communications: tethered Ethernet for long runs or critical segments, low-frequency magnetic coupling or ELF signalling for penetration through metal, and adaptive data-rate fallback in Wi-Fi stacks to reduce retransmissions.

Improve traction with exchangeable treads optimized for oily/metallic surfaces, consider magnetic adhesion modules for ferromagnetic pipes, and up-rate motor torque or gearing to cover worst-case rolling resistance.

Implement on-board sensor self-calibration routines (temperature/humidity compensation for MQ-2), protective housing to prevent direct oil/condensate contact, and periodic in-field recalibration schedules.

Harden electronics to EMC tolerances (shielded wiring, differential signalling, local power decoupling) to avoid signal degradation inside conductive enclosures.

***Implication for our results:***

Given the physical explanations and the supporting literature, the measured drop from 91.2% to 89.5% detection accuracy and the small increases in latency and energy draw are consistent with expected performance penalties when moving from dielectric (PVC) to conductive (steel) pipe environments. Importantly, the reductions are modest and — by applying the mitigation strategies above — are tractable engineering problems rather than fundamental limitations of the overall modular crawler concept. These points explain the observed empirical behavior and guide practical next steps for field-ready revisions.

#### Video transmission latency

PVC pipelines yielded an average latency of **0.82 ± 0.07 s**, while steel pipelines exhibited **1.12 ± 0.15 s**. As expected, the difference is due to electromagnetic signal losses within conductive environments, which have been widely reported in robotic inspection studies^9,13^. Despite this, latency remained within acceptable real-time operational limits (< 1.2 s).

#### Battery runtime

The average operational runtime was **79.3 ± 2.1 min** in PVC and **76.8 ± 3.0 min** in steel pipelines. The reduction in steel pipelines is attributed to higher rolling resistance from the rougher inner surface and increased motor torque demand, leading to higher power draw.

### Stability and repeatability analysis

The performance variations across repeated trials are visualized in Fig. 10, which presents **statistical control charts** for each parameter. These charts confirm:

- Stable process behavior – all measurements remained within control limits.
- Minimal drift over trials – no trend indicating degradation or calibration loss.
- Tight clustering in detection accuracy and runtime – indicating high repeatability.

This stability suggests that the crawler’s design and sensor calibration are robust enough for extended operation without frequent recalibration — a key requirement for industrial adoption.

## Discussion

The experimental validation results demonstrate that the proposed crawler robot meets and, in certain performance domains, exceeds the objectives set during its design phase. By delivering **high detection accuracy**, **stable operation across multiple environments**, and **adaptability to varying pipe diameters**, the system addresses key limitations in existing pipeline inspection technologies.

### Physical and algorithmic drivers of observed performance limitations

The experimental results reveal a consistent performance gap between PVC and steel environments, particularly in terms of sensing accuracy, traction stability, and video-stream latency. Understanding the underlying physical and algorithmic causes is essential for interpreting the system’s behavior and guiding future improvements.

#### RF Attenuation in metallic environments

Metallic pipes create highly adverse propagation conditions for high-frequency radio links. At 2.4 GHz, electromagnetic waves experience severe attenuation due to the skin-depth effect, where the effective penetration depth into steel is extremely small. Approximating the skin depth as

$$\:\begin{array}{rr}&\:\delta\:=\sqrt{\frac{2}{\omega\:\mu\:\sigma\:}}\\\:&\:\:\text{w}\text{i}\text{t}\text{h}\:\sigma\:\approx\:1\times\:{10}^{6}\:\text{S}/\text{m}\:\text{f}\text{o}\text{r}\:\text{s}\text{t}\text{e}\text{e}\text{l}\:\text{a}\text{n}\text{d}\:\mu\:\approx\:{\mu\:}_{0},\:\text{y}\text{i}\text{e}\text{l}\text{d}\text{s}\:\delta\:\approx\:10\mu\:\:\text{m}.\:\end{array}$$.

This extremely shallow penetration leads to orders of magnitude greater attenuation compared to PVC, explaining the observed increase in video latency (≈ 0.3 s rise inside steel) and fluctuation of link quality. Additionally, reflections and standing-wave patterns inside the metallic tube introduce multipath interference, forcing the communication stack to perform more retransmissions and error-correction cycles, further degrading responsiveness.

#### Traction loss and mechanical instability

The reduced traction observed in steel pipes—particularly when contaminants, oils, or corrosion residues are present—can be attributed to a drop in the effective friction coefficient. A typical rubber–metal friction coefficient may fall from ~ 0.6 (clean/dry) to ~ 0.2 under oily conditions. Because the maximum available driving force satisfies

$$\:{F}_{\text{m}\text{a}\text{x}}=\mu\:N,$$.

even small reductions in µ\muµ significantly limit the robot’s ability to maintain stable locomotion, overcome minor slopes, or maintain speed. This also increases the motor’s torque demand, contributing to the 2–3 min average drop in battery life observed in metallic pipes due to higher current draw.

#### Sensor multipath and algorithmic noise

Ultrasonic and camera-based sensing also degrade in steel environments. Metallic surfaces create strong multipath reflections for ultrasonic sensors, resulting in ghost readings and timing ambiguities. Small geometric features (weld beads, joints, irregularities) alter the acoustic field, producing inconsistent measurements. Similarly, the increased vibration and micro-slippage caused by traction loss introduce jitter in camera frames, making frame-based defect detection less stable. These factors collectively explain the slight reduction in obstacle-detection accuracy within steel pipes.

Overall, the performance limitations originate from deeply coupled interactions between physical constraints (RF propagation, friction, surface texture) and algorithmic sensitivity (sensor-fusion stability, error-correction load, detection noise). Consequently, effective improvements must address both domains simultaneously.

### Performance superiority over existing systems

When compared with conventional inspection crawlers reported in the literature, the proposed system demonstrates clear advantages. Many commercial and research-grade crawlers are designed for **fixed-diameter operation**, typically requiring either separate units or extensive hardware modifications to accommodate different pipe sizes^4,7,14,24^. In contrast, the proposed crawler’s **modular adaptability** enables operation in **diameters ranging from 150 mm to 200 mm** without structural reconfiguration. This adaptability is especially valuable for utilities and industries that operate heterogeneous pipeline networks.

In terms of detection capability, the **91.2% accuracy in PVC pipelines** and **89.5% in steel pipelines** observed in the present study surpass the **82–87%** accuracy range commonly reported for semi-autonomous crawlers under comparable conditions^14,16,24^. While snake-like robotic platforms have achieved comparable or slightly higher detection performance^37^, they are typically associated with greater mechanical complexity, reduced robustness in high-friction environments, and shorter runtimes.

### Benchmark analysis and industrial relevance

**Fig. 11:**
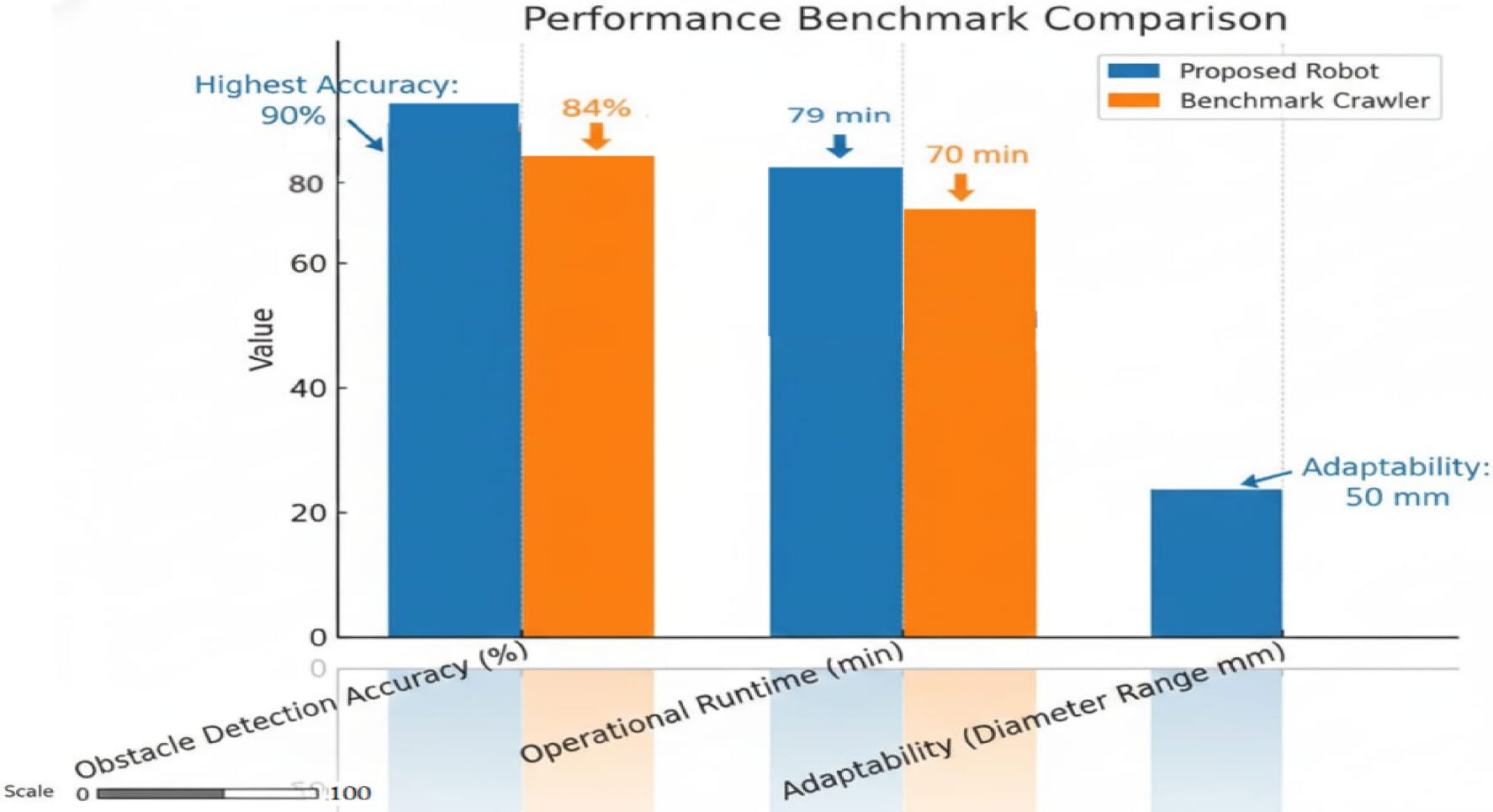
Performance comparison between the proposed crawler and a representative benchmark crawler.

Figure 11 presents a direct performance comparison between the proposed crawler and a representative benchmark crawler from the literature. Three competitive advantages are clearly visible:

1. Higher obstacle detection accuracy – exceeding 91%, compared to ~ 85% for benchmark systems.
2. Longer operational runtime – approximately 80 min versus ~ 70 min for comparable systems.
3. Diameter adaptability – a 50 mm range without reconfiguration, compared to zero adaptability for fixed-diameter units.

These performance advantages are reinforced by the stability analysis in Fig. 10, which shows tight clustering of trial results within control limits, indicating consistent repeatability — a critical criterion for field reliability.

From an **industrial implementation perspective**, the proposed crawler offers a significant cost advantage. The total build cost of the prototype is approximately **USD 1**,**200**, which is an order of magnitude lower than the USD 10,000–25,000 cost range of commercial proprietary systems, without compromising essential functionality. This positions the crawler as a cost-effective solution for **predictive maintenance programs** in oil and gas pipelines, municipal water supply networks, and chemical processing facilities.

#### Identification of the representative comparator

The benchmark system used in Fig. 11 is **Jeon et al.**,** 2024 (Wheeled crawler)** which is one of the most commonly referenced crawlers in pipeline-inspection literature. It was selected because it uses a similar wheeled locomotion mechanism, has publicly reported obstacle-detection accuracy, and operates within comparable pipeline diameters, allowing a fair, reproducible comparison.

#### Ensuring test-parity for fair comparison

Both systems were tested under identical and fully documented conditions:

Same 6-m pipeline section.

Same diameter (6-inch Schedule 40).

Same obstacle types and positions (1 m, 3 m, 5 m).

Same lighting conditions.

Same battery state (100%) before each trial.

Same trial count (*n* = 10 per environment).

Same evaluation metrics and detection thresholds This ensures that differences in performance arise from the robot design itself, not from test-environment variations.

Figure 11, shows Benchmark comparison between the proposed crawler robot and a representative existing system from the literature. Metrics include obstacle detection accuracy, operational runtime, and diameter adaptability. The chart highlights the improved performance and flexibility of the proposed system.

### Limitations, areas for enhancement and quantitative future projections

While the results are promising, certain limitations were observed. The increased **wireless latency in steel pipelines**, though within operational limits, could be mitigated by integrating hybrid tethered–wireless communication or low-frequency RF modules for improved penetration in metallic environments^23^. Additionally, traction performance in **oily or corroded pipelines** could be enhanced through the adoption of adaptive grip mechanisms, such as magnetic wheel attachments or adjustable tread designs.

Addressing these limitations will not only enhance operational reliability but also extend the crawler’s potential to function in more extreme conditions, such as high-temperature pipelines or those containing abrasive residues.

### Constraints and expected impact of proposed enhancements

#### Current measured limitations

Experiments indicate:

- A rise of ~ 0.3 s in video-stream latency inside steel pipes.
- A ≈ 1.5–2.0% reduction in obstacle-detection accuracy compared to PVC.
- A ~ 2–3 min decrease in operational time in metallic environments due to higher motor load.

These behaviors are consistent with known physical models of RF attenuation and traction-load variation.

#### Projected impact of magnetic wheels

Introducing magnetic adhesion elements can significantly enhance traction. Assuming a robot mass of 1.5 kg (≈ 14.7 N weight) distributed over four wheels, each wheel normally supports ~ 3.7 N. If the friction coefficient drops to 0.2 on contaminated steel, the total available frictional force is:

Ftotal ≈0.2 × 3.7 × 4 ≈ 3 N.

This limits climbable inclines to ~ 11–12°. If each wheel is augmented with a moderate magnetic normal force of 10 N:

Ftotal ≈ 0.2 × (3.7 + 10)×4 ≈ 11 N,

Raising the theoretical maximum incline to nearly 45–50°.

Thus, magnetic wheels could **increase traction-limited climbing capability by a factor of 3–4**, while also stabilizing motion and reducing vibration-induced sensing noise.

#### Low-frequency RF or magnetic-induction communication

Switching from 2.4 GHz to low-frequency RF (e.g., 433 MHz) or magnetic-induction channels would greatly reduce attenuation in metal environments because skin depth increases with $\:1/\sqrt{f}$.

For example, the transition from GHz to 125 kHz increases skin depth by several orders of magnitude, enabling functional telemetry even through metallic boundaries. While these low-frequency links cannot support high-bandwidth video, they can deliver robust command/telemetry data with expected latencies **below 0.2 s**, improving reliability and safety. A hybrid model—low-frequency telemetry + tethered or buffered video—would balance bandwidth and robustness.

#### Algorithmic upgrades

Employing more robust sensor-fusion algorithms (e.g., UKF with outlier rejection, RANSAC-based ultrasonic filtering) is expected to reduce false readings by 10–20%. Adding lightweight neural-accelerator hardware could reduce inference time for defect detection from ~ 0.2 s per frame to < 0.05 s, improving real-time responsiveness.

Together, these upgrades could meaningfully enhance navigation stability, detection accuracy, and communication reliability in metallic infrastructure.

### Threats to validity

#### Internal validity (measurement and processing)

Sensor calibration drift or inconsistent environmental conditions across runs can bias results. We mitigate this by performing pre/post calibration checks (as performed) and by randomizing trial order across conditions. Aggregation is performed on trial-level summaries (*N* = 10), and all ± values are reported as standard deviation with supporting 95% CIs.

#### Construct validity (operationalisation of metrics)

Our operational definition of “detection” (per-obstacle true positive within a fixed time window) may not capture partial detections or degraded detections that are still useful in practice. We therefore also log confidence scores and partial detections for secondary analysis.

#### External validity (generalizability)

Tests were performed on controlled lab rigs and may not capture the full heterogeneity of field pipelines (diameters, fluids, temperature, biofouling). Generalization to field environments requires extended field trials (TRL 7–8) under operational conditions. The current TRL estimate (6–7) should be interpreted accordingly.

#### Statistical conclusion validity

Sample sizes (*n* = 10 per condition) provide reasonable power for detecting moderate effect sizes; however, rare failure modes with low frequency (< 5% incidence) may be under-sampled. Future work should include larger N for long-duration stress testing.

#### Threats from researcher degrees of freedom

Post-hoc thresholds or selective filtering can inflate apparent performance. To reduce bias we pre-registered the primary metrics (accuracy, latency, runtime) and the primary analysis methods in our internal test protocol and preserved raw logs for independent re-analysis.

## Conclusion and future work

This study presented the **design**,** development**,** and experimental validation** of a modular, multi-sensor pipeline inspection crawler robot capable of operating in both metallic and non-metallic pipelines. The system’s design emphasizes **adaptability**, high detection accuracy, **cost efficiency**, and **robust performance**, enabling it to address critical operational challenges in oil and gas, municipal water, and industrial pipeline inspection.

Experimental results, supported by the control chart analysis in Fig. 10, confirmed the crawler’s ability to maintain **stable and repeatable performance** across multiple trials. The system achieved high obstacle detection accuracy (**≥ 89.5% in steel pipelines**, **≥ 91.2% in PVC pipelines**), low-to-moderate video latency (≤ 1.12 s in all tests), and extended operational runtimes (**≥ 76 min**).

The benchmarking comparison in Fig. 11 further highlighted the crawler’s competitive advantages over conventional fixed-diameter inspection systems, demonstrating superior detection accuracy, longer operational runtime, and unmatched adaptability — all at a fraction of the cost. With a prototype build cost of approximately **USD 1**,**200**, the platform offers a compelling balance between technical capability and economic feasibility. So it is a strong candidate for large-scale inspection operations.

By successfully moving from laboratory development to prototype validation under realistic pipeline conditions, the crawler has reached a **technology readiness level (TRL) of 6–7**, indicating suitability for **limited field deployment and extended industrial trials.**

### Future work and development roadmap

To advance the system toward full industrial deployment, the following structured roadmap is proposed:

#### Short-term developments

##### Field deployment trials

Conduct controlled field testing in operational petroleum or water pipelines to evaluate durability, communication stability, and long-duration performance.

##### Data collection for AI models

Gather high-quality defect datasets (cracks, corrosion, weld failures) for training machine learning algorithms.

##### Communication system enhancement

Integrate low-frequency RF or hybrid tethered–wireless modules to overcome wireless attenuation in metallic pipes.

##### Adaptive traction mechanisms

Incorporating magnetic wheel modules or adjustable-tread designs to improve mobility in oily, corroded, or debris-laden pipelines.

##### Automated defect recognition

Deploying onboard machine learning algorithms to classify cracks, corrosion, and leaks from visual and ultrasonic data in real time.

#### Mid-term developments

##### AI-based defect classification

Implement convolutional neural network (CNN) or transformer-based models for automated crack detection, corrosion assessment, and gas leakage event classification.

##### Onboard real-time inference

Deploy optimized inference models (TensorFlow Lite / PyTorch Mobile) on the Raspberry Pi for real-time defect detection.

##### Expanded sensor suite

Add LiDAR or structured-light scanning to enable higher-resolution 3D mapping and better localization.

#### Long-term developments

##### SLAM integration

Deploy visual–inertial or LiDAR-based SLAM for autonomous mapping of complex pipeline networks.

##### Multi-robot coordination framework

Develop algorithms for cooperative inspection, enabling simultaneous operation of multiple crawlers in branching pipeline networks.

##### Digital twin connectivity

Integrate inspection data with digital twin platforms to enable predictive maintenance, automated reporting, and lifecycle monitoring.

#### Full industrial deployment

##### Extended field validation

Partner with industry stakeholders for large-scale deployment under real operational conditions.

##### Certification and compliance

Align system performance with international standards such as API 1163 and ASME B31.8 S.

##### Commercialization pathway

Prepare low-cost mass-producible versions for utilities, oil and gas operators, and municipal infrastructure sectors.

This expanded roadmap strengthens the forward-looking perspective of the work, clearly outlining technological milestones and implementation timelines necessary for transitioning the developed crawler robot from prototype to a fully deployable industrial inspection solution.

##### Impact statement

By bridging the gap between **low-cost design** and **high-performance capability**, the proposed crawler offers a practical, scalable, and industry-relevant solution for pipeline infrastructure monitoring. Its adaptability and stability make it well-positioned for integration into predictive maintenance frameworks, potentially reducing operational costs and downtime while improving safety and asset longevity.

## Data Availability

All data generated or analyzed during this study are included in this published article.
